# Serendipitous Conversion of an Acetylamino Dideoxy-Octonic Acid Derivate into a Functionalized Carbohydrate–Pyrazole Conjugate and Investigation of the Method´s General Applicability

**DOI:** 10.3390/molecules29204885

**Published:** 2024-10-15

**Authors:** Jelena K. Berl, Christian Czaschke, Ann-Kathrin Pramor, Christian B. W. Stark, Joachim Thiem

**Affiliations:** Department of Chemistry, Institute for Organic Chemistry, University of Hamburg, Martin-Luther-King-Platz 6, D-20146 Hamburg, Germany; jelena.berl@uni-hamburg.de (J.K.B.); christian.czaschke@studium.fernuni-hagen.de (C.C.); ann-kathrin.pramor@uni-hamburg.de (A.-K.P.)

**Keywords:** pyrazole synthesis, ADOA, sialic acid derivate

## Abstract

By treatment of the peracetylated methylester of 4-acetylamino-2,4-dideoxy-d-glycero-d-galacto-octonic acid (ADOA-PAE) with nitrosyl tetrafluoroborate, a serendipitous formation of a highly functionalized carbohydrate–pyrazole conjugate was observed in 95% yield. This observation is remarkable, as it involves a five-step one-pot synthesis that proceeds via an 1,3-acyl shift and a 1,5-electrocyclization, which usually requires thermal conditions; however, the reaction occurred at a temperature of 0 °C. Additionally, the excellent yield of the carbohydrate-decorated pyrazole and the regiospecificity of the cyclization are of particular interest, as regioselectivity is always a challenge in pyrazole synthesis. Subsequently, this novel access to pyrazoles starting from *N*-acetyl-allyl amides via nitrosation and electrocyclization was investigated. In addition, mechanistic studies for the formation of substituted pyrazoles of type were carried out.

## 1. Introduction

**Figure 1:**
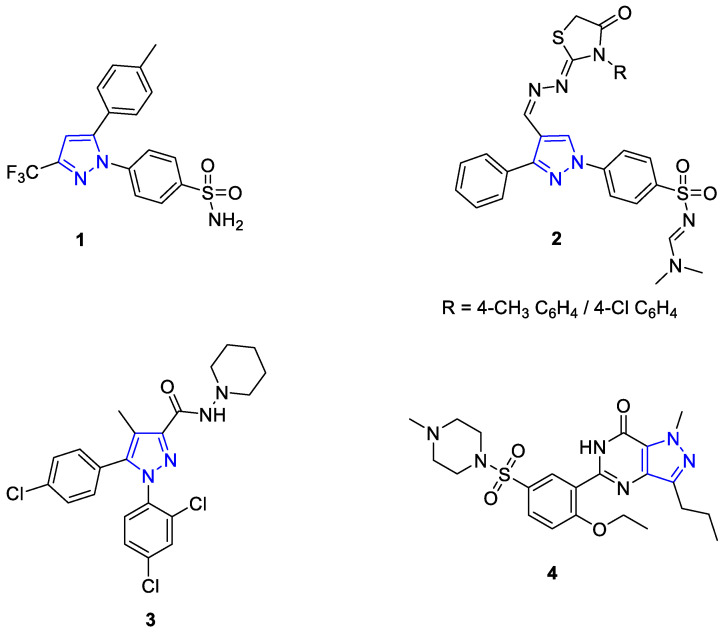
Structure of the biologically active compounds celecoxib (**1**), compound (**2**), rimonabant (**3**) and sildenafil (**4**) [11,12,13,14,15].

Pyrazoles represent a class of heterocycles with a broad range of applications. Although they are rare in nature due to a lack of *N*-*N*-bond-forming enzymes, pyrazoles are found as substructures in biologically active substances such as drugs and agrochemicals, or they are used as ligands in coordination chemistry [1,2,3,4,5,6]. The syntheses of pyrazoles is an ongoing topic, so that current methods have further developed or even overcome the conventional cyclocondensation and cycloaddition chemistry [7,8,9,10]. Pyrazole-containing molecules are of particular interest in the field of non-steroidal anti-inflammatory drugs (NSAIDs), because they are known as selective cyclooxygenase-2 (COX-2) inhibitors, which can lead to reduced side effects compared to steroids. For example, celecoxib (**1**) and compound (**2**) have an improved gastrointestinal tolerance [11]. Other pharmaceuticals that contain a pyrazole core include rimonabant (**3**) and sildenafil (**4**) (Figure 1) [12]. Rimonabant (**3**) acts as an antagonist at the cannabinoid-1 receptor (CB-1). Since the endocannabinoid system is directly linked to the consumption of sweet and high-fat foods, it was used as an appetite suppressant [13]. Sildenafil (**4**) acts as a phosphodiesterase-5 inhibitor. Phosphodiesterase is an enzyme that breaks down cyclic guanosine monophosphate (cGMP). An increase in cGMP concentration leads to muscle relaxation and vasodilation. Consequently, sildenafil citrate is marketed as a pharmaceutical for the treatment of erectile dysfunction under the brand name Viagra [14,15]. 

**Scheme 1:**
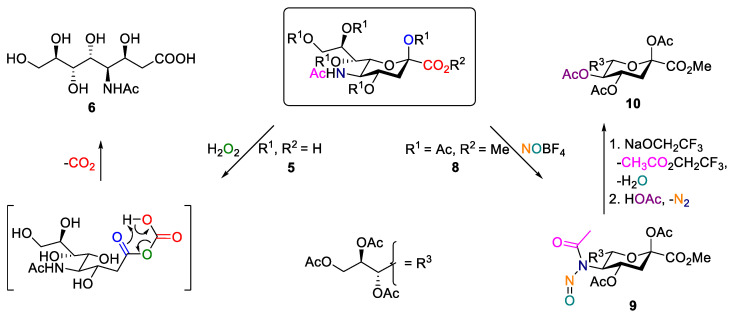
The preparation of 4-acetylamino-2,4-dideoxy-d-glycero- d-galacto-octonic acid (ADOA, **6**) starting from *N*-acetylneuraminic acid (Neu5Ac, **5**) as well as the synthesis of the peracetylated methyl ester of 3-deoxy-d-glycero-d-galacto-nonulosonic acid (**10**) starting from the peracetylated methyl ester of Neu5Ac (**8**) [20,21,22].

As a part of our general interest in reactions of *N*-acetylneuramic acid (**5**) [16,17,18] and its derivative 4-acetylamino-2,4-dideoxy-d-glycero-d-galacto-octonic acid (**6**, ADOA) [19], the nitrosation of peracetylated acyclic ester of ADOA (**7**, ADOA-PAE) was investigated. The synthesis of ADOA (**6**) was performed as previously described as a H_2_O_2_-induced Bayer–Villiger oxidation of neuraminic acid, which is followed by methyl esterification and the subsequent acetylation of the hydroxyl groups [20]. Similar to Zibral et al. and Crich et al., the formation of the corresponding *N*-nitroso compound was expected [21,22]. Zibral et al. described the conversion of the peracetlyated methylester of *N*-acetylneuraminic acid (**8**) by nitrosation to the *N*-nitroso compound (**9**), which could subsequently be converted through the addition of sodium trifluoroethanolate via the corresponding diazo compound, which is followed by the addition of acetic acid to the peracetylated methyl ester of 2-keto-3-deoxy-d-glycero-d-galacto-nononic acid (**10**) (Scheme 1) [20].

**Scheme 2:**
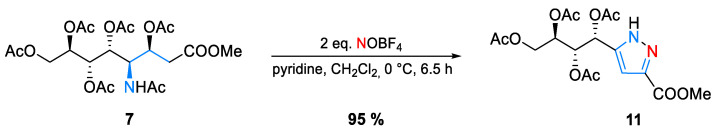
Formation of pyrazole (**11**) starting from ADOA-PAE (**7**).

During the nitrosation of ADOA-PAE (**7**), we were surprised to isolate not the anticipated *N*-nitroso product but instead a highly functionalized carbohydrate–pyrazole conjugate (**11**) with a 95% yield. Thus, after initial nitrosation, the rapid elimination of acetic acid furnished unsaturated diazo intermediate, which underwent an 1,5-electrocyclization (Scheme 2).

## 2. Results

**Scheme 3:**
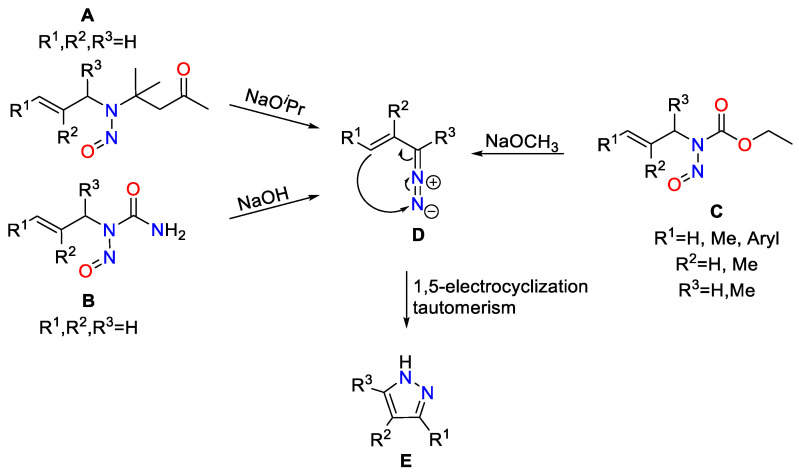
Known syntheses of pyrazoles starting from *N*-nitroso compounds [27,28,29,30].

It is well known that the treatment of *N*-nitroso-allylamino*iso*butyl methyl ketone (**A**), *N*-nitroso-*N*-allylurea (**B**) or *N*-nitroso-*N*-allyl carbamates (**C**) with base leads to intermediary vinyldiazoalkanes (**D**), which in turn rearrange to form pyrazoles (**E**) (Scheme 3) [23,24,25,26]. In general, 1,5-electrocyclizations of vinyl diazo compounds (**D**) have been described as a versatile means for the regiospecific preparation of pyrazoles [27,28,29,30]. 

However, the accessibility of vinyl diazoalkanes is a limiting factor and hampers the broad application of this method. As described by Kirmse, a synthetic approach for the preparation of diazo compounds is the alkali cleavage of *N*-nitroso amides or the thermal rearrangement to diazo esters followed by subsequent deprotonation (Scheme 3) [31]. To the best of our knowledge, this approach has never been used for reactant generation in pyrazole synthesis [32,33]. In contrast to Kirmse’s conditions, a less strong base (pyridine) was present, and no thermal conditions were applied.

**Scheme 4:**
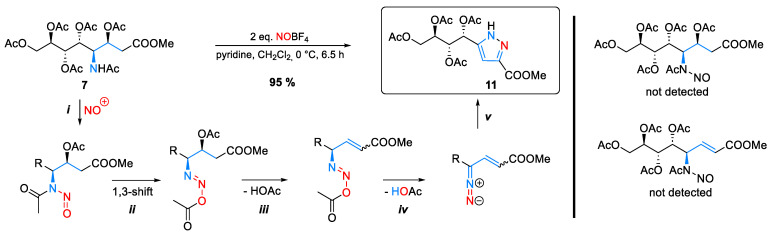
Formation of pyrazole (**11**) starting from ADOA-PAE (**7**) in a one-pot five step synthesis.

The exceptionally high yield and the striking efficiency of this one-pot five step synthesis consisting of nitrosation (***i***), 1,3-acyl shift (***ii***), elimination of acetic acid (***iii***), deprotonation (***iv***), and 1,5-electrocyclization (***v***) prompted us to investigate the general applicability of this reaction in more detail (Scheme 4).

**Scheme 5:**
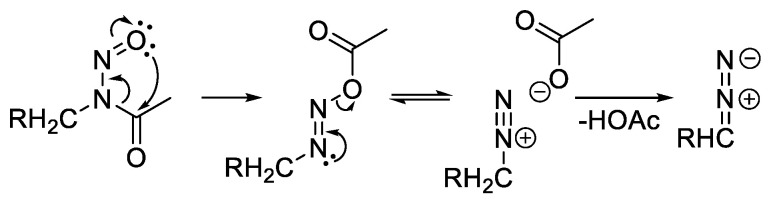
1,3-Acyl shift of *N*-nitroso amides and formation of diazo compounds via deprotonation [33].

In order to investigate the generality of the pyrazole synthesis described in Scheme 2, we decided to move away from the carbohydrate backbone and significantly reduced the complexity of the substrate structure. With respect to simplicity and accessibility, simple *N*-acetyl allyl amides were identified as a minimal motif and used as starting materials. Therefore, one step less (elimination of acetic acid) is required in the overall process from the *N*-acetyl amide to the pyrazole (as compared to the reaction sequence summarized in Scheme 5).

Based on these considerations, the nitrosation of a series of *N*-acetyl allylic amines (**12a–d**) under similar conditions as for ADOA-PAE (**7**) was performed. Allylamides (**12b**) and (**12c**) were prepared by hydrazinolysis and the subsequent acetylation of phthalimides (**22b**) and (**22c**). The simplification of the structure led to *N*-acetyl-allyl amine (**12a**) as the minimal motif. However, it was found that two consecutive operations were required. For complete conversion in the nitrosation reaction, a higher concentration than in the conversion of (**7**) was required. In this process, initially, *N*-acetyl-*N*-nitroso allyl amines (**13a–d**) were obtained instead of the respective 1*H*-pyrazoles (**14a–d**). Due to the volatility and instability of *N*-nitroso amides, the intermediate compounds were filtered over silica gel, which is a solvent that has been largely removed under reduced pressure and further reacted directly with DBU. Identification of the resulting *N*-nitroso compounds was based on their characteristic green colors, their *R*_f_ values as well as the *N*-acetyl shifts observed in ^1^H-NMR. The yields over two steps, starting from the *N*-acetyl-allyl amines (**12a–d**), were moderate for 1*H*-pyrazole (**14a**) as well as for 3(5)-propyl-pyrazole (**14b**). In contrast, only low yields were obtained for 3(5)-methyl-pyrazole (**14c**) and 4-methyl-pyrazole (**14d**) (Table 1).

As indicated by moderate to low yields, this approach does not seem to provide a synthetically useful method for the preparation of pyrazoles. The extraordinarily high yield of pyrazole (**11**) and the fact that pyridine was sufficient as a base for in situ deprotonation, whereas DBU was required for substrates (**13a–d**), emphasized the peculiarity of substrate (**7**) and led to particular interest in the mechanism involved.

In order to obtain insight into the structural basis for the high efficiency of the process with respect to substrate (**7**) and to clarify the role of the carboxylate, it was decided to test substrate (**15a**), which represents a substructure of the elimination product of ADOA-PAE (**7**). By nitrosating (**15a**), the corresponding *N*-nitroso amide (**16a**) could be obtained. However, the reaction with DBU to give the respective diazoalkane in case of (**16a**) led to decomposition.

Therefore, it was decided to exchange the strong base DBU for the milder base pyridine like in our original finding. Compared to the conversion of ADOA-PAE (**7**), the reaction temperature was varied instead of the base. Thus, the 1,5-electrocyclization was tested under thermal conditions at 40 °C, and the corresponding pyrazole (**17a**) could be isolated in 14% yield. Although the cyclization proceeded successfully, the yield was much lower than for the preparation of pyrazole (**11**). Subsequently, substrate (**15b**), which carries one more CH_2_OAc group compared to (**15a**) and thus comes closer to the structure of ADOA-PAE (**7**), was prepared. When DBU was used to initiate cyclization, decomposition was observed. On the other hand, thermal conditions again led to the formation of pyrazole (**17b**) in a yield of 15%. 

Finally, substrate (**15c**) was tested, which mainly differs from substrate (**15b**) in a (*Z*)-configured double bond. Using DBU, pyrazole (**17c**) was obtained in a 21% yield, whereas under thermal conditions, a good yield of 63% was obtained (Table 2).

Subsequently, a temperature (23 °C, 40 °C, 60 °C) and solvent (CH_2_Cl_2_, CDCl_3_, 1,2-DCE) screening for the conversion of substrate (**15c**) to pyrazole (**17c**) was carried out (Table 3). A comparably good yield of 60% was observed for cyclization in CDCl_3_ at 40 °C, whereas at the same temperature in CH_2_Cl_2_, only a 15% yield was obtained. In all solvents, hardly any conversion was observed at room temperature; at 60 °C, decomposition already occurred.

**Figure 2:**
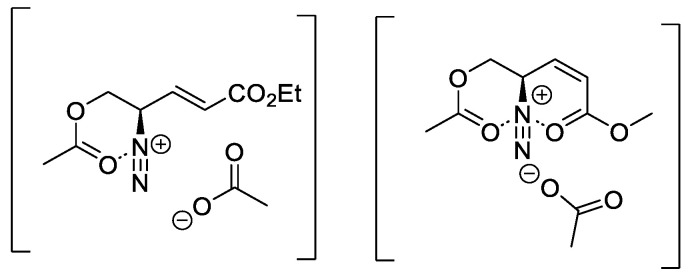
Stabilization of in situ generated diazonium ion by donor function of acetate/acetate and ester.

Finally, method B was applied to substrates (**12a–d**), but no conversion could be observed. It can be concluded that the alkyl substituted substrates (**12a–d**) could only be reacted with strong bases such as DBU in moderate yields. Carbonyl substituted substrates (**15a–c**), on the other hand, could be converted exclusively or in better yields with mild bases like pyridine at higher temperatures. In case of compounds (**16a–c**), a stabilizing charge dipole interaction of the diazonium ion formed in situ with the carbonyl oxygen of the ester substituents is suggested (Figure 2). 

This effect is particularly prominent in the case of two ester functions and a (*Z*)-configured double bond as in substrate (**16c**). It can be assumed that in addition to this effect, one or more acetate groups of ADOA-PAE (**7**) are involved in catalyzing the 1,3-acyl-shift. Thus, the rearrangement will already occur at 0 °C, and no thermal conditions of 40 °C are required as in the case of substrates (**16a–c**). 

**Scheme 6:**
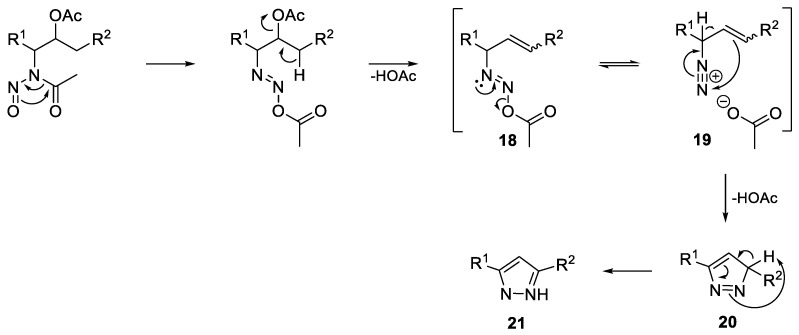
Proposed mechanism of pyrazole formation.

Furthermore, since the pKa values of substrates (**13a–e**) are assumed to be significantly higher, a strong base such as DBU is necessary for deprotonation of the intermediate diazonium ion. The low yields can easily be explained by the extrusion of nitrogen as a competing reaction, which is followed by the known carbocation chemistry. By summarizing the above results, the mechanism for pyrazole formation is proposed in Scheme 6. Following a 1,3-acyl shift, the elimination of acetic acid gives the resulting diazoacetate (**18**). This is in equilibrium with diazonium ion (**19**), which after deprotonation undergoes a 6π electrocyclization to give 3*H*-pyrazole (**20**) followed by tautomerization to 1*H*-pyrazole (**21**).

## 3. Conclusions

The above findings support the assumption that the formation of pyrazole (**11**) from ADOA-PAE (**7**) by nitrosation followed a five-step domino reaction [34,35]. Studies to investigate the features of this remarkable transformation led to two novel protocols for the synthesis of pyrazoles. Thus, simple alkyl substituted and carbonyl substituted *N*-nitroso allyl amides could be converted into pyrazoles in moderate to good yields, and mechanistic interpretations are suggested.

## 4. Experimental Procedure

### 4.1. General

All reagents were purchased from commercial suppliers and used as received. Nitrosyl tetrafluoroborate was predried for 30 min at 10^−1^ mbar before use. TLC was carried out on Merck silica gel 60 F_254_ plates. Compounds were detected using UV light and/or by staining with a potassium permanganate reagent and subsequent heating. The staining reagent was composed of potassium permanganate (2.4 g), potassium carbonate (16.0 g), sodium hydroxide solution (4 mL, 5%, aq) and water (240 mL). Column chromatography was performed with Merck/Fluka silica gel 60 (230–400 mesh). Solvents for column chromatography were distilled prior to use. ^1^H and ^13^C NMR spectra (Supplementary Materials) were recorded with Bruker FourierHD 300 MHz, Bruker AMX-400, Bruker AV-400, Bruker AV-500 or AV-600 spectrometers (Bruker, Karlsruhe, Germany); spectra were calibrated using the residual solvent peak. Melting points were measured with a Büchi melting point M-565 (Büchi, Flawil, Switzerland). Optical rotations were obtained at 589 nm using a Krüss Optronic P8000 polarimeter (Krüss, Hamburg, Germany). HRMS (ESI-TOF) spectra were recorded with a Agilent 6224 ESI-TOF mass spectrometer (Agilent, Waldbronn, Germany), and EI spectra were recorded with a Thermo ISQ LT EI mass spectrometer (Thermo, Bremen, Germany). IR spectra were recorded on a Bruker ALPHA FT-IR Platinum ATR (Bruker, Karlsruhe, Germany). Wave numbers are reported in reciprocal centimeters (cm^−1^). 

Due to the instability of the nitroso compounds, they could not be characterized by MS spectrometry. Pyrazoles were identified by NMR and MS data and compared to the literature. *N*-Nitroso compounds were characterized and, in a separate experiment, used directly for the preparation of pyrazoles with yields determined over two steps.

### 4.2. Syntheses

#### 4.2.1. General Procedure A for Nitrosation 

A round-bottom flask was charged with amide (1.0 equiv.), anhydrous dichloromethane (*c*_Amide_ = 0.6 mol/L) and anhydrous pyridine (10 equiv.), cooled to 0 °C, and nitrosyl tetrafluoroborate (1.5 equiv.) was then added. The reaction mixture was stirred for 5 h at 0 °C; then, the suspension was filtered over silica gel with dichloromethane and concentrated.

#### 4.2.2. General Procedure B for Nitrosation 

A round-bottom flask was charged with amide (1.0 equiv.), anhydrous dichloromethane (*c*_Amide_ = 0.1 mol/L) and anhydrous pyridine (10 equiv.), cooled to 0 °C, and then nitrosyl tetrafluoroborate (2.0 equiv.) was added. The reaction mixture was stirred for 5 h at 0 °C; then, the suspension was filtered over silica gel with dichloromethane and concentrated. 

#### 4.2.3. General Procedure for Base Induced Cyclization 

A round-bottom flask was charged with *N*-nitroso compound (1.0 equiv. based on amide), anhydrous THF (*c*_NO-compound_ = 0.6 mol/L) and DBU (1.0 equiv.). The reaction mixture was stirred at room temperature until complete conversion was obtained; then, it was dissolved in ethyl acetate, filtered over silica gel and concentrated. 

#### 4.2.4. General Procedure of Heat-Induced Cyclization 

A round-bottom flask was charged with *N*-nitroso compound (1.0 equiv. based on amide), anhydrous 1,2-dichloroethane (*c*_NO-compound_ = 0.1 mol/L) and anhydrous pyridine (*c*_NO-compound_ = 1.3 mol/L). The reaction mixture was heated to reflux for 15 h, concentrated and purified by column chromatography.

#### 4.2.5. (1*R*,2*S*,3*R*)-1-(3-(Methoxycarbonyl)-1*H*-pyrazol-5-yl)butane-1,2,3,4-tetrayl tetraacetate (**11**)

Pyrazole **11** was isolated by the general procedure B for the nitrosation of ADOA-PAE (**7**, 0.275 mmol) after column chromatographic purification (petrol ether/ethyl acetate 1:1) as white crystals in a yield of 95% (0.261 mmol). Mp: 124.4 °C, [α]_D_^20^ = −41 (*c* = 0.3, CH_2_Cl_2_); HRMS (ESI) *m*/*z*: for [M-OAc]^+^ calc for C_15_H_19_N_2_O_8_^+^: 355.1136, found: 355.1128. IR (ATR) ν: = 1739, 1703, 1373, 1212, 1174, 1056, 1030, 1001, 973, 958, 854, 814, 776, 611, 509, 479, 397 cm^−1^. ^1^H-NMR (400 MHz, CDCl_3_) δ: 10.84 (bs, 1H, NH), 6,81 (s, 1H, H-3), 6.20 (d, *J_5,6_* = 4.1 Hz, 1H, H-5), 5.62 (dd, *J_5,6_* = 4.1, *J_6,7_* = 7.8 Hz, 1H, H-6), 5.24 (ddd, *J_6,7_* = 8.0, *J_7,8a_* = 2.9, *J_7,8b_* = 5.3 Hz, 1H, H-7), 4.27 (dd, *J_7,8a_* = 3.0, *J_8a,8b_* = 12.4 Hz, 1H, H-8_a_), 4.15 (dd, *J_7,8b_* = 5.3, *J_8a,8b_* = 12.4 Hz, 1H, H-8_b_), 3.91 (s, 3H, CO_2_C**H_3_**), 2.12, 2.08, 2.05, 2.04 (s, 4xOCOC**H_3_**, 12H). ^13^C-NMR (126 MHz, CDCl_3_) δ: 170.8, 170.0, 170.0, 169.5 (O**C**OCH_3_), 159.9 (C-1), 147.7 (C-4), 136.5 (C-2), 107.8 (C-3), 70.7 (C-6), 68.7 (C-7), 67.2 (C-5), 61.9 (C-8), 52.5 (COO**C**H_3_), 20.9, 20.8, 20.8, 20.7 (4xOCO**C**H_3_).

#### 4.2.6. Hydrazinolysis and Acetylation

To a solution of phthalimide (1.0 equiv.) in ethanol (*c*_phthalimide_ 0.67 mol/L) was added hydrazine hydrate (51%, 2.0 equiv.). The suspension was stirred at 50 °C for 1 h; then, dichloromethane was added, and the suspension was filtered. The filter cake was rinsed with dichloromethane, the filtrate was washed with water, and the aqueous phase was extracted three times with dichloromethane. The combined organic phases were dried over sodium sulfate and filtered. Triethylamine (4.5 equiv.) and acetic anhydride (5.0 equiv.) were added to the combined organic phases, and the resultant solution was stirred for 48 h at room temperature. The reaction mixture was washed with saturated ammonium chloride solution, and the aqueous phase was extracted three times with dichloromethane. The combined organic phases were dried over magnesium sulfate, filtered, and concentrated.

#### 4.2.7. *N*-Acetyl-allyl amine (**12a**)

The product was prepared using a procedure in the literature [36]. The crude product was purified by vacuum distillation (10 mbar/100 °C) to give 80% (11 mmol) of a colorless liquid. 

#### 4.2.8. (*E*)-2-(Hex-2-en-1-yl)isoindoline-1,3-dione (**22b**)

To a solution of (*E*)-bromohex-2-ene (3.24 mmol, 1.0 equiv.) in *N*,*N*-dimethylformamide (*c*_alkyl halide_ = 0.64 mol/L) was added potassium phthalimide (1.1 equiv). The suspension was stirred at 50 °C for 2 h and then extracted with water and dichloromethane. The aqueous phase was extracted three times with dichloromethane; then, the combined organic layers were washed with 0.2 M sodium hydroxide solution, dried over sodium sulfate, filtered and concentrated. The crude product was purified by column chromatography on silica gel (petrol ether/ethyl acetate 50:1) to give 2.17 mmol (67%) of (*E*)-2-(hex-2-en-1-yl)isoindoline-1,3-dione (**22b**) as a white amorphous solid. Mp: 36.8 °C, HRMS (ESI) *m*/*z*: for [M+H]^+^ calc for C_14_H_16_NO_2_^+^: 230.1176; found 230.1179. IR(ATR) ν: = 3463, 2956, 2926, 1768, 1710, 1392, 1347, 1333, 1298, 1268, 1138, 1087, 1068, 1037, 999, 934, 711, 623, 528. ^1^H-NMR (400 MHz, CDCl_3_): δ = 7.86–7.82 (m, 2H, H-3), 7.73–7.68 (m, 2H, H-4), 5.74 (dtt, *J_5,7_* = 1.3, *J_6,7_* = 14.9, *J_7,8_* = 6.7 Hz, 1H, H-7), 5.51 (dtt, *J_5,6_* = 6.2, *J_6,7_* = 15.4, *J_6,8_* = 1.5 Hz, 1H, H-6), 4.23 (dd, *J_5,6_* = 6.4, *J_5,7_* = 1.1 Hz, 2H, H-5), 1.98 (dtt, *J_6,8_* = 1.2, *J_7,8_* = 6.7, *J_8,9_* = 5.6 Hz, 2H, H-8), 1.37 (sext, *J_8,9_*/*J_9,10_* = 7.4 Hz, 2H, H-9), 0.86 (t, *J_9,10_* = 7.4 Hz, 3H, H-10). ^13^C-NMR (126 MHz, CDCl_3_): δ = 168.2 (C-1), 135.2 (C-7), 134.0 (C-4), 132.4 (C-2), 123.4 (C-6), 123.4 (C-3), 39.8 (C-5), 34.3 (C-8), 22.2 (C-9), 13.8 (C-10).

#### 4.2.9. (*E*)-N-(Hex-2-en-1-yl)Acetamide (**12b**)

(*E*)-2-(Hex-2-en-1-yl)isoindoline-1,3-dione (**22b**, 2.02 mmol) was used as phthalimide. The crude product was purified by column chromatography on silica gel with ethyl acetate to give 1.65 mmol (82%) of (*E*)-*N*-acetylhex-2-enylamide (**12b**) as a colorless liquid. MS (EI): *m*/*z* (%): 141 (8) [M^+^], 112 (13) [M-(CH_2_CH_3_)^+^], 70 (100) [M-(CH_3_CO)- (CH_2_CH_3_)^+^], 43 (56) [(CH_3_CO)^+^]. IR (ATR) ν: = 3280, 2959, 2928, 1649, 1545, 1457, 1432, 1373, 1282, 1243, 967, 735, 601 cm^−1^. ^1^H-NMR (400 MHz, CDCl_3_): δ = 5.60 (dtt, *J_1,3_* = 1.4, *J_2,3_* = 14.7, *J_3,4_* = 6.6 Hz, 1H, H-3), 5.53 (s, 1H, NH), 5.43 (dtt, *J_1,2_* = 6.2, *J_2,3_* = 15.3, *J_2,4_* = 1.4 Hz, 1H, H-2), 3.80 (td, *J_1,2_* = 5.9, *J_1,3_* = 1.3 Hz, 2H, H-1), 2.07–1.93 (m, 5H, H-4, H-8), 1.37 (sext, *J_4,5_*/*J_5,6_* = 7.4 Hz, 2H, H-5), 0.88 (t, *J_5,6_* = 7.4 Hz, 3H, H-6). ^13^C-NMR (101 MHz, CDCl_3_): δ = 169.9 (C-7), 133.9 (C-3), 125.8 (C-2), 41.8 (C-1), 34.4 (C-4), 23.4 (C-8), 22.4 (C-5), 13.8 (C-6). 

#### 4.2.10. (*E*/*Z*)-2-(But-2-en-1-yl)isoindoline-1,3-dione (**22c**)

To a solution of (*E*/*Z*)-crotylchloride (3.92 mmol, *E*:*Z* 6:1, 70%, 1.0 equiv.) in *N*,*N*-dimethylformamide (*c*_alkyl halide_ = 0.64 mol/L) was added potassium phthalimide (1.1 equiv). The suspension was stirred at 50 °C for 2 h and then extracted with water and dichloromethane. The aqueous phase was extracted three times with dichloromethane; then, the combined organic layers were washed with 0.2 M sodium hydroxide solution, dried over sodium sulfate, filtered and concentrated. The crude product was purified by column chromatography on silica gel (PE:EA 4:1) to give 90% (3.52 mmol, *E*:*Z* 6:1) of (*E*/*Z*)-2-(But-2-en-1-yl)isoindoline-1,3-dione (**22c**) as a white amorphous solid. Mp: 71.4 °C, HRMS (ESI) *m*/*z*: for [M+H]^+^ calc for C_12_H_12_NO_2_^+^: 202.0863; found 202.0859. IR(ATR) ν: = 2919, 1708, 1465, 1426, 1391, 1354, 1330, 1081, 1064, 795, 613, 527 cm^−1^. ^1^H-NMR (400 MHz, CDCl_3_): δ [ppm] = 7.87–7.80 (m, 4H, H-3_E,Z_), 7.73–7.67 (m, 4H, H-4_E,Z_), 5.80–5.63 (m, 2H, H-7_E,Z_), 5.59-5.44 (m, 2H, H-6_E,Z_), 4.32 (ddd, *J_5,6_* = 7.0, *J_5,7_* = 1.5, *J_5,8_* = 0.8 Hz, 2H, H-5_Z_), 4.22 (dp, *J_5,6_* = 6.2, *J_5,7_*/*J_5,8_* = 1.2 Hz, 2H, H-5_E_), 1.82 (ddt, *J_5,8_* = 0.8, *J_6,8_* = 1.8, *J_8,7_* = 6.9 Hz, 3H, H-8_Z_), 1.67 (dq, *J_6,8_*/*J_5,8_* = 1.3, *J_7,8_* = 6.5 Hz, 3H, H-8_E_). ^13^C-NMR (126 MHz, CDCl_3_): δ [ppm] = 168.2 (C-1_E,Z_), 134.0 (C-4_E,Z_), 132.4 (C-2_E,Z_), 130.0 (C-7_E,Z_), 124.6 (C-6_E,Z_), 123.4 (C-3_E,Z_), 39.6 (C-5_E_), 34.7 (C-5_Z_), 17.7 (C-8_E_), 13.1 (C-8_Z_).

#### 4.2.11. (*E*/*Z*)-*N*-(But-2-en-1-yl)acetamide (**12c**)

(*E*/*Z*)-2-(But-2-en-1-yl)isoindoline-1,3-dione (3.52 mmol, **22c**) was used as phthalimide. The crude product was purified by column chromatography on silica gel (petrol ether/ethyl acetate 2:1) to give 1.95 mmol (55%, E:Z 6:1) of (*E*/*Z*)-*N*-acetylcrotylamine (**3c**) as a colorless liquid. MS (EI): *m*/*z* (%): 113 (15) [M^+^], 70 (46) [M-(CH_3_CO)^+^], 56 (100) [(C_3_H_6_N)^+^], 43 (56) [(CH_3_CO)^+^]. IR (ATR) ν: = 3282, 2919, 1648, 1542, 1434, 1373, 1281, 1099, 1040, 1004, 964, 712, 629, 481 cm^−1^. ^1^H-NMR (600 MHz, CDCl_3_): δ = 5.66–5.58 (m, 2H, H-3_E,Z_), 5.53 (bs, 2H, NH_E,Z_), 5.48–5.37 (m, 2H, H-2_E,Z_), 3.91–3.87 (m, 2H, H-1_Z_), 3.80–3.75 (m, 2H, H-1_E_), 1.97 (s, 6H, H-6_E,Z_), 1.69–1.66 (m, 6H, H-4_E,Z_). ^13^C-NMR (101 MHz, CDCl_3_): δ = 169.9 (C-5_E,Z_), 128.7 (C-3_E_), 128.1 (C-3_Z_), 127.0 (C-2_E_), 126.0 (C-2_Z_), 41.7 (C-1_E_), 36.6 (C-1_Z_), 23.4 (C-6_E,Z_), 17.8 (C-4_E_), 13.1 (C-4_Z_).

#### 4.2.12. *N*-(2-Methylallyl)-acetamide (**12d**)

Compound **12d** was prepared using a procedure in the literature [36]. The crude product was purified by distillation (7 mbar/120 °C) to give 0.71 mmol (65% based on 2-methylallylamine) of a colorless liquid.

#### 4.2.13. *N*-Allyl-*N*-nitroso acetamide (**13a**)

Compound **13a** was prepared starting from *N*-allylacetamide (**12a**, 1.49 mmol) according to the general procedure A for nitrosation. The product was obtained as a green liquid. IR (ATR) ν: = 1733, 1438, 1374, 1241, 1114, 1045, 925, 882, 747, 703, 660, 603, 406 cm^−1^. ^1^H-NMR (500 MHz, CDCl_3_): δ = 5.56 (ddt, *J_1_,_2_* = 5.8, *J_2,3-trans_* = 17.1, *J_2,3-cis_* = 10.2 Hz, 1H, H-2), 5.10 (dq, *J_2,3-cis_* = 10.2, *J_3-cis,3-trans_*/*J_1,3-cis_* = 1.2 Hz, 1H, H-3_cis_), 5.05 (dq, *J_2,3-trans_* = 17.1, *J_3-trans,3-cis_*/*J_1,3-trans_* = 1.4 Hz, 1H, H-3_trans_), 4.36 (dt, *J_1,2_* = 5.7, *J_1,3_* = 1.4 Hz, 2H, H-1), 2.79 (s, 3H, H-5). ^13^C-NMR (126 MHz, CDCl_3_): δ = 174.2 (C-4), 129.3 (C-2), 118.7 (C-3), 40.8 (C-1), 22.6 (C-5). Due to instability, MS analysis could not be performed.

#### 4.2.14. (*E*)-*N*-Nitroso-*N*-acetylhex-2-enylamine (**13b**)

The compound was prepared starting from (*E*)-*N*-acetylhex-2-enylamine (0.69 mmol, **12b**) according to the general procedure A for nitrosation. The product was obtained as a green liquid. IR (ATR) ν: = 2960, 2931, 1729, 1503, 1418, 1375, 1336, 1111, 949, 883, 776, 642, 514, 423 cm^−1^. ^1^H-NMR (500 MHz, CDCl_3_): δ = 5.58 (dtt, *J_2,3_* = 14.9, *J_3,4_* = 6.8, *J_1,3_* = 1.3 Hz, 1H, H-3), 5.18 (dtt, *J_1,2_* = 6.4, *J_2,3_* = 15.5, *J_2,4_* = 1.5 Hz, 1H, H-2), 4.29 (dd, *J_1,2_* = 6.3, *J_1,3_* = 1.1 Hz, 2H, H-1), 2.76 (s, 3H, H-8), 1.91 (q, *J_3,4_*/*J_4,5_* = 7.2 Hz, 2H, H-4), 1.33 (sext, *J_5,4_*/*J_5,6_* = 7.4 Hz, 2H, H-5), 0.83 (t, *J_5,6_* = 7.4 Hz, 3H, H-6). ^13^C-NMR (126 MHz, CDCl_3_): δ = 174.3 (C-7), 136.4 (C-3), 121.1 (C-2), 40.4 (C-1), 34.2 (C-4), 22.7 (C-8), 22.1 (C-5), 13.7 (C-6). Due to instability, MS analysis could not be performed.

#### 4.2.15. (*E*/*Z*)-*N*-Nitroso-*N*-acetylcrotylamine (**13c**)

The compound was prepared starting from *N*-(but-2-en-1-yl)acetamide (**12c**, 0.44 mmol) according to the general procedure A for nitrosation. The product was obtained as a green liquid. IR (ATR) ν: = 2943, 2922, 1728, 1500, 1374, 1109, 951, 920, 881, 641 cm^−1^. ^1^H-NMR (600 MHz, CDCl_3_): δ = 5.60 (dqt, *J_1,3_* = 1.3, *J_2,3_* = 15.7, *J_3,4_* = 6.5 Hz, 2H, H-3_E,Z_), 5.21 (dtq, *J_1,2_* = 6.3, *J_2,3_* = 16.1, *J_2,4_* = 1.7 Hz, 1H, H-2_E_), 5.12-5.06 (m, 1H, H-2_Z_), 4.40 (d, *J_1,2_* = 6.9, 2H, H-1_Z_), 4.30–4.26 (m, 2H, H-1_E_), 2.77 (s, 6H, H-6_E,Z_), 1.76–1.73 (m, 3H, H-4_Z_), 1.61 (dq, *J_2,4_*/*J_1,4_* = 1.3, *J_3,4_* = 6.5 Hz, 3H, H-4_E_). ^13^C-NMR (151 MHz, CDCl_3_): δ = 174.3 (C-5_E,Z_), 131.2 (C-3_E_), 129.8 (C-3_Z_), 122.3 (C-2_E_), 121.7 (C-2_Z_), 40.4 (C-1_E_), 36.0 (C-1_Z_), 22.7 (C-6_E,Z_), 17.7 (C-4_E_), 13.1 (C-4_E_). Due to instability, MS analysis could not be performed.

#### 4.2.16. *N*-(2-Methylallyl)-*N*-nitrosoacetamide (**13d**)

The compound was prepared starting from **12d** (0.44 mmol) according to the general procedure A for nitrosation. The product was obtained as a green liquid. IR (ATR) ν: = 2922, 2851, 1736, 1630, 1556, 1449, 1387, 1340, 1322, 1290, 1242, 1156, 1109, 950, 930, 891, 862, 833, 817, 753, 696, 619, 597, 518, 490 cm^−1^. ^1^H-NMR (400 MHz, CDCl_3_): δ = 4.78–4.72 (m, 1H, H-1_a_), 4.49–4.43 (m, 1H, H-1_b_), 4.28 (bs, 2H, H-3), 2.81 (s, 3H, H-6), 1.64 (dd, *J_1a,4_* = 1.6, *J_1b,4_* = 0.8 Hz, 3H, H-4). ^13^C-NMR (126 MHz, CDCl_3_): δ = 174.3 (C-5), 137.2 (C-2), 111.7 (C-1), 43.7 (C-3), 22.6 (C-6), 20.5 (C-4). Due to instability, MS analysis could not be performed.

#### 4.2.17. 1*H*-pyrazole (**14a**)

To raw **13a** dissolved in dichloromethane (2.6 mL) and distilled water (0.20 mL), DBU (3.0 mmol, 2.0 equiv.) was added. The reaction mixture was stirred for 20 h at room temperature and then filtered over silica gel with ethyl acetate. The filtrate was concentrated to yield 43% (0.64 mmol) over 2 steps of a white amorphous solid. MS (EI): *m*/*z* (%): 68 (100) [C_3_H_4_N_2_^+^], 41 (23) [C_3_H_5_^+^]. IR (ATR) ν: = 3122, 3048, 2854, 1465, 1394, 1356, 1133, 1030, 873, 753, 615. ^1^H-NMR (500 MHz, CDCl_3_): δ [ppm] = 7.63 (d, *J_3,4_*/*J_4,5_* = 2.1 Hz, 2H, H-3, H-5), 6.36 (t, *J_3,4_*/*J_4,5_* = 2.1 Hz, 1H, H-4). ^13^C-NMR (126 MHz, CDCl_3_): δ [ppm] = 133.8 (C-3, C-5), 105.2 (C-4). The spectroscopic data correspond to values in the literature [37].

#### 4.2.18. 3(5)-Propyl-1H-pyrazole (**14b**)

Pyrazole **14b** was synthesized by the general procedure for base-induced cyclization starting from **13b**. The crude product was purified by column chromatography on silica gel with ethyl acetate to give 0.16 mmol (23%) of **14b** as a white amorphous solid. MS (EI): *m*/*z* (%): 110 (27) [(M)^+^], 81 (100) [(C_4_H_5_N_2_)^+^], 54 (8) [(C_2_H_2_N_2_)^+^], 41 (9) [(C_2_H_3_N)^+^]. IR (ATR) ν: = 3185, 2960, 2931, 2872, 1465, 1370, 1342, 1107, 1051, 936, 872, 804, 767 cm^−1^. ^1^H-NMR (400 MHz, CDCl_3_): δ = 7.49 (d, *J_4,5_* = 2.0 Hz, 1H, H-5), 6.08 (d, *J_4,5_* = 2.0 Hz, 1H, H-4), 2.66 (t, *J_6,7_* = 7.6 Hz, 2H, H-6), 1.69 (sext, *J_7,6_*/*J_7,8_* = 7.4 Hz, 2H, H-7), 0.97 (t, *J_7,8_* = 7.3 Hz, 3H, H-8). ^13^C-NMR (101 MHz, CDCl_3_): δ = 147.8 (C-3), 135.2 (C-5), 103.5 (C-4), 28.8 (C-6), 22.8 (C-7), 14.0 (C-8). The spectroscopic data correspond to values in the literature [38].

#### 4.2.19. 3(5)-Methyl-1*H*-pyrazole (**14c**)

Pyrazole **14c** was synthesized by the general procedure for base-induced cyclization starting from **13c**. The non-UV active product could not be visualized by staining agents. Thus, the concentration was determined by quantitative NMR and the yield calculated based on anisole as internal standard. The crude product was obtained as a yellow solid in a yield of 12% (0.05 mmol). MS (EI): *m*/*z* (%): 82 (100) [M^+^], 54 (26) [(C_4_H_5_)^+^], 40 (7) [(C_3_H_4_)^+^]. ^1^H-NMR (300 MHz, CDCl_3_): δ = 7.48 (d, *J_4,5_* = 2.0 Hz, 1H, H-5), 6.08 (d, *J_4,5_* = 2.0 Hz, 1H, H-4), 2.34 (s, 3H, H-6). ^13^C-NMR (126 MHz, CDCl_3_): δ = 143.6 (C-3), 135.0 (C-5), 104.6 (C-4), 12.2 (C-6). The spectroscopic data correspond to values in the literature [39].

#### 4.2.20. 4-Methyl-1*H*-pyrazole (**14d**)

Pyrazole **14d** was isolated by the general procedure for base-induced cyclization starting from **13d**. The non-UV active product could not be visualized by staining agents, the concentration was determined by quantitative NMR, and the yield was calculated based on 1,3,5-trimethoxybenzene as an internal standard. The crude product was obtained as a brown solid in a yield of 5% (20 μmol). ^1^H-NMR (500 MHz, CDCl_3_): δ = 7.33 (s, 2H, H-3, H-5), 2.08 (s, 3H, H-6). ^13^C-NMR (75 MHz, CDCl_3_): δ = 132.9 (C-3, C-5), 115.2 (C-4), 8.8 (C-6). The spectroscopic data correspond to values in the literature [39].

#### 4.2.21. (*E,Z*)-4-*N*-Acetamido but-2-Enoic Acid Ethylester (**15a**)

To a solution of *N*-allyl acetamide (**12a**) (2.02 mmol, 1.0 equiv.) in anhydrous CH_2_Cl_2_ (20 mL) was added ethyl acrylate (20.2 mmol, 10 equiv.). Then, 2nd Generation Grubbs Hoveyda Catalyst (0.14 mmol, 7.0 mol%) was added, and the suspension was refluxed for 48 h. The reaction mixture was concentrated under reduced pressure, and the crude product was purified by column chromatography on silica gel with ethyl acetate to give 66% (1.33 mmol, *E*:*Z* 25:1) of a red liquid. HRMS (ESI) *m*/*z*: for [M+Na]^+^ calc for C_8_H_14_NO_3_^+^: 172.0968, found: 172.0966. IR (ATR) ν: = 3289, 2982, 1716, 1651, 1540, 1368, 1271, 1176, 1036, 971, 730, 682, 600 cm^−1^. ^1^H-NMR (600 MHz, CDCl_3_): δ = 6.85 (dt, *J_2,3_* = 15.7, *J_3,4_* = 5.1 Hz, 1H, H-3_E_), 6.44 (s, 1H, NH_Z_), 6.33 (s, 1H, NH_E_), 6.22 (dt, *J_2,3_* = 11.5, *J_3,4_* = 6.3 Hz, 1H, H-3_Z_), 5.87 (dt, *J_2,3_* = 15.7, *J_2,4_* = 1.9 Hz, 1H, H-2_E_), 5.82 (dt, *J_2,3_* = 11.5, *J_2,4_* = 1.8 Hz, 1H, H-2_Z_), 4.30 (td, *J_3,4_*/*J_4,NH_* = 6.3, *J_2,4_* = 1.9 Hz, 2H, H-4_Z_) 4.14 (q, *J_2,4_* = 7.1 Hz, 4H, H-7_E,Z_), 3.98 (td, *J_3,4_*_/_ *J_4,NH_* = 5.5, *J_2,4_* = 1.9 Hz, 2H, H-4_E_), 1.99 (s, 3H, H-6_E_), 1.95 (s, 3H, H-6_Z_), 1.24 (t, *J_8,7_* = 7.1 Hz, 6H, H-8_E,Z_). ^13^C-NMR (151 MHz, CDCl_3_): δ = 170.1 (C-5_E_), 166.1 (C-1_E_), 143.9 (C-3_E_), 134.3 (C-2_Z_), 122.1 (C-2_E_), 60.7 (C-7_E,Z_), 40.4 (C-4_E_), 37.8 (C-4_Z_), 23.4 (C-6_Z_), 23.3 (C-6_E_), 14.4 (C-8_E,Z_). Due to the low concentration in the sample, C-1_Z_, C-3_Z_ and C-5_Z_ could not be assigned.

#### 4.2.22. Ethyl(*E*)-3-[(*R*)-3-(tert-butoxycarbonyl)-2,2-dimethyloxazolidine-4-yl]propenoate (**23b**)

The product was prepared using a procedure in the literature [40]. The crude product was purified by column chromatography on silica gel (petrol ether/ethyl acetate 7:3) to give 84% (0.82 mmol) of a white, amorphous solid.

#### 4.2.23. Ethyl (*R,E*)-4-acetamido-5-acetoxypent-2-enoate (**15b**)

To a solution of **23b** (1.0 equiv.) in ethanol (*c*_S1/S2_ = 0.14 mol/L) was added *p*-toluenesulfonic acid monohydrate (1.5 equiv.), and the solution was refluxed. After 5 h, triethylamine (1.5 equiv.) was added, and the reaction mixture was concentrated under reduced pressure. The crude product was dissolved in dichloromethane (c = 0.05 mol/L), and acetic anhydride (4.5 equiv.) and triethylamine (4.5 equiv.) were added. After stirring for 2 days at room temperature, the reaction mixture was washed with saturated ammonium chloride solution, and the aqueous phase was extracted three times with dichloromethane. The combined organic phases were dried over magnesium sulfate, filtered, and concentrated. The crude product was purified by column chromatography on silica gel (petrol ether/ethyl acetate 3:7) twice to give 52% (0.11 mmol) of a white, amorphous solid. Mp: 104.9–106.5 °C, [α]_D_^20^ = −5.6 (*c* = 0.47, CH_2_Cl_2_); HRMS (ESI) *m*/*z*: for [M+Na]^+^ calc for C_11_H_17_NNaO_5_^+^: 266.0999, found: 266.0998. IR (ATR) ν: = 3280, 2980, 1722, 1650, 1547, 1385, 1303, 1185, 1154, 1040, 983, 779, 623, 615, 490, 422 cm^−1^. ^1^H-NMR (500 MHz, CDCl_3_): δ = 6.83 (dd, *J_2,3_* = 15.8, *J_3,4_* = 4.9 Hz, 1H, H-3), 5.97 (dd, *J_2,3_* = 15.7, *J_2,4_* = 1.5 Hz, 1H, H-2), 5.90 (d, *J_4,NH_* = 7.9 Hz, 1H, NH), 4.99–4.90 (m, 1H, H-4), 4.26 (dd, *J_4,5a_* = 5.2, *J_5a,5b_* = 11.4 Hz*,* 1H, H-5_a_), 4.20 (q, *J_10,11_* = 7.2 Hz, 2H, H-10), 4.15 (dd, *J_4,5b_* = 3.9, *J_5a,5b_* = 11.2 Hz, 1H, H-5_b_), 2.08 (s, 3H, H-9), 2.05 (s, 3H, H-7), 1.29 (t, *J_10,11_* = 7.1 Hz, 3H, H-11). ^13^C-NMR (126 MHz, CDCl_3_): δ = 171.1 (C-8), 169.8 (C-6), 165.9 (C-1), 143.4 (C-3), 123.3 (C-2), 65.2 (C-5), 60.9 (C-10), 49.6 (C-4), 23.4 (C-7), 20.9 (C-9), 14.3 (C-11).

#### 4.2.24. (*R*)-Tert-Butyl 4-{[(*Z*)-2-methoxycarbonyl]vinyl}-2,2-dimethyloxazolidine-3-car-boxylate (**23c**)

The product was prepared using a procedure literature [41]. The crude product was purified by column chromatography on silica gel (petrol ether/ethyl acetate 9:1) to give 60% (1.8 mmol) of a white, amorphous solid.

#### 4.2.25. Methyl (*R,Z*)-4-Acetamido-5-acetoxypent-2-enoate (**15c**)

To a solution of **23c** (1.0 equiv.) in methanol (*c*_S1/S2_ = 0.14 mol/L) was added *p*-toluenesulfonic acid monohydrate (1.5 equiv.), and the solution was refluxed. After 5 h, triethylamine (1.5 equiv.) was added, and the reaction mixture was concentrated under reduced pressure. The crude product was dissolved in dichloromethane (c = 0.05 mol/L), and acetic anhydride (4.5 equiv.) and triethylamine (4.5 equiv.) were added. After stirring for 2 days at room temperature, the reaction mixture was washed with saturated ammonium chloride solution, and the aqueous phase was extracted three times with dichloromethane. The combined organic phases were dried over magnesium sulfate, filtered, and concentrated. The crude product was purified by column chromatography on silica gel (petrol ether/ethyl acetate 3:7–100% ethyl acetate) to give 61% (0.38 mmol) of a white, amorphous solid. Mp: 60.6–63.1 °C, [α]_D_^16^ = 20.9 (*c* = 0.11, CH_2_Cl_2_); HRMS (ESI) *m*/*z* for C_10_H_15_NO_5_ (229.0950): [M+Na]^+^: calc: 252.0842, found: 252.0842. IR (ATR) ν: = 3287, 3086, 2960, 1718, 1640, 1547, 1435, 1405, 1371, 1322, 1230, 1205, 1071, 822, 745, 606 cm^−1^. ^1^H-NMR (500 MHz, CDCl3): δ = 6.43 (d, *J_4,NH_* = 7.7 Hz, 1H, NH), 6.13 (dd, *J_2,3_* = 11.6, *J_3,4_* = 7.9 Hz, 1H, H-3), 5.92 (dd, *J_2,3_* = 11.7, *J_2,4_* = 1.3 Hz, 1H, H-2), 5.53 (ddddd, *J_4,NH_* = 7.7, *J_3,4_* = 7.7, *J_4,5a_* = 5.6, *J_4,5b_* = 4.2 Hz, *J_2,4_* = 1.3 Hz, 1H, H-4), 4.34 (dd, *J_4,5a_* = 6.2, *J_5a,5b_* = 11.3 Hz, 1H, H-5_a_), 4.22 (dd, *J_4,5a_* = 4.2, *J_5a,5b_* = 11.3 Hz, 1H, H-5_b_), 3.74 (s, 3H, H-10), 2.08 (s, 3H, H-9), 1.98 (s, 3H, H-7). ^13^C-NMR (126 MHz, CDCl3): δ [ppm] = 171.4 (C-8), 169.9 (C-6), 166.2 (C-1), 145.8 (C-3), 122.2 (C-2), 65.5 (C-5), 51.8 (C-10), 48.3 (C-4), 23.5 (C-7), 21.0 (C-9).

#### 4.2.26. (*E*)-4-*N*-Acetamido-*N*-nitrosobut-2-enoic Acid Ethylester (**16a**)

The compound was prepared starting from **15a** (0.29 mmol) according to the general procedure A for nitrosation. The product was obtained as a green liquid. IR (ATR) ν: = 2983, 2924, 1716, 1505, 1401, 1373, 1309, 1181, 1038, 943, 900, 640 cm^−1^. ^1^H-NMR (500 MHz, CDCl_3_): δ = 6.59 (dt, *J_2,3_* = 15.7, *J_3,4_* = 5.5 Hz, 1H, H-3), 5.68 (dt, *J_2,3_* = 15.8, *J_2,4_* = 1.7 Hz, 1H, H-2), 4.49 (dd, *J_2,4_* = 1.7, *J_3,4_* = 5.6 Hz, 2H, H-4), 4.16 (q, *J_7,8_* = 7.1 Hz, 2H, H-7), 2.82 (s, 3H, H-6), 1.26 (t, *J_7,8_* = 7.1 Hz, 3H, H-8). ^13^C-NMR (126 MHz, CDCl_3_): δ = 173.8 (C-5), 165.5 (C-1), 138.2 (C-3), 124.1 (C-2), 60.8 (C-7), 38.9 (C-4), 22.6 (C-6), 14.3 (C-8). Due to instability, MS analysis could not be performed.

#### 4.2.27. Ethyl (*R,E*)-5-Acetoxy-4-(*N*-nitroso-acetamido)pent-2-enoate (**16b**)

The compound was prepared starting from **15b** (0.82 μmol) according to the general procedure B for nitrosation. The product was obtained as a green liquid. IR (ATR) ν: = 2983, 1718, 1650, 1368, 1221, 1184, 1035, 928, 736, 705, 654, 604, 527 cm^−1^. ^1^H-NMR (300 MHz, CDCl_3_): δ = 6.79 (dd, *J_2,3_* = 15.9, *J_3,4_* = 6.0 Hz, 1H, H-3), 5.76 (dd, *J_2,3_* = 15.9, *J_2,4_* = 1.7 Hz, 1H, H-2), 5.61 (dddd, *J_2,4_* = 1.7, *J_3,4_* = 5.9, *J_4,5a_* = 6.0 Hz, *J_4,5b_* = 7.8 Hz, 1H, H-4), 4.41 (dd, *J_4,5a_* = 5.9, *J_5a,5b_* = 11.9 Hz, 1H, H-5_a_), 4.25 (dd, *J_4,5b_* = 8.3, *J_5a,5b_* = 11.3 Hz, 1H, H-5_b_), 4.15 (q, *J_10,11_* = 7.1 Hz, 2H, H-10), 2.75 (s, 3H, H-7), 1.95 (s, 3H, H-9), 1.24 (t, *J_10,11_* = 7.1 Hz, 3H, H-11). ^13^C-NMR (75 MHz, CDCl_3_): δ = 174.3 (C-6), 170.2 (C-8), 165.3 (C-1), 139.0 (C-3), 124.7 (C-2), 61.7 (C-5), 60.9 (C-10), 49.6 (C-4), 23.2 (C-7), 20.6 (C-9), 14.2 (C-11). Due to instability, MS analysis could not be performed.

#### 4.2.28. Methyl (*R,Z*)-5-Acetoxy-4-(*N*-nitroso-acetamido)pent-2-enoate (**16c**)

The compound was prepared starting from **15c** (0.10 mmol) according to the general procedure B for nitrosation. The product was obtained as a green liquid. IR (ATR) ν: = 2955, 1721, 1647, 1525, 1372, 1222, 1199, 1180, 1123, 1039, 929, 825, 705, 639, 602 cm^−1^. ^1^H-NMR (300 MHz, CDCl_3_): δ = 6.43–6.26 (m, 2H, H-3, H-4), 5.84 (d, *J_2,3_* = 9.9 Hz, 1H, H-2), 4.32–4.10 (m, 2H, H-5), 3.66 (s, 3H, H-10), 2.73 (s, 3H, H-7), 1.96 (s, 3H, H-9). ^13^C-NMR (75 MHz, CDCl_3_): δ = 174.5 (C-6), 170.4 (C-8), 165.3 (C-1), 139.7 (C-3), 123.0 (C-2), 62.2 (C-5), 51.8 (C-10), 47.3 (C-4), 23.2 (C-7), 20.7 (C-9). Due to instability, MS analysis could not be performed.

#### 4.2.29. Ethyl-1*H*-pyrazole-3-carboxylate (**17a**)

The compound was prepared from **16a** according to the general heat-induced cyclization. The crude product was purified by column chromatography on silica gel (petrol ether/ethyl acetate 2:1) to give 40 μmol (14%) of **17a** as a white amorphous solid. IR (ATR) ν: = 3243, 2923, 1697, 1373, 1300, 1222, 1177, 1050, 1019, 835, 816, 761, 626, 421. ^1^H-NMR (500 MHz, CDCl_3_): δ = 7.76 (d, *J*_3,4_ = 2.3 Hz, 1H, H-4), 6.93–6.84 (m, 1H, H-3), 4.43 (q, *J*_5,6_ = 7.1 Hz, 2H, H-5), 1.41 (t, *J*_5,6_ = 7.1 Hz, 3H, H-6). The ^1^H-NMR signal of the NH was not visible in the solvent used. ^13^C-NMR (150 MHz, DMSO-d_6_): δ [ppm] = 162.2 (C-1), 142.9 (C-2), 130.0 (C-4), 107.4 (C-3), 60.0 (C-5), 14.3 (C-6). HRMS (ESI) *m*/*z* for C_6_H_8_N_2_O_2_ (140.0586): [M+Na]^+^: calc: 163.0478, found: 163.0485. The spectroscopic data correspond to the values in the literature [42].

#### 4.2.30. Ethyl 5-(Acetoxymethyl)-1*H*-pyrazole-3-carboxylate (**17b**)

The compound was prepared from **16b** according to the general method of heat-induced cyclization. Pyrazole **17b** was isolated after column chromatographic purification (petrol ether/ethyl acetate 4:1) as yellow oil in a yield of 15% (11 μmol). HRMS (ESI) *m*/*z*: for [M+Na]^+^ calc for C_9_H_12_N_2_NaO_4_^+^: 235.0689; found 235.0687. ^1^H-NMR (500 MHz, CDCl_3_): δ = 6.86 (s, 1H, H-3), 5.14 (s, 2H, H-5), 4.39 (q, *J_8,9_ =* 7.1 Hz, H-8), 2.11 (s, 3H, H-7), 1.39 (t, *J_8,9_* = 7.1 Hz, H-9). ^13^C-NMR (126 MHz, CDCl_3_): δ = 171.4 (C-6), 160.2 (C-1), 144.7 (C-4), 138.4 (C-2), 109.0 (C-3), 61.6 (C-8), 58.6 (C-5), 21.0 (C-7), 14.4 (C-9). The spectroscopic data correspond to the values in the literature [43].

#### 4.2.31. Methyl 5-(Acetoxymethyl)-1*H*-pyrazole-3-carboxylate (**17c**)

The compound was prepared from **16c** according to the general method of heat induced cyclization. Pyrazole **17c** was isolated after column chromatographic purification (petrol ether/ethyl acetate 4:1) as a white, amorphous solid in a yield of 63% (0.064 mmol). Using the general method for base-induced cyclization, pyrazole (**17c**) was obtained in a yield of 21% (0.030 mmol). HRMS (ESI) *m*/*z*: for [M+Na]^+^ calc for C_8_H_10_N_2_NaO_4_^+^: 221.0533, found: 221.0516. ^1^H-NMR (500 MHz, CDCl_3_): δ = 6.86 (s, 1H, H-3), 5.15 (s, 2H, H-5), 3.92 (s, 3H, H-6), 2.10 (s, 3H, H-8). ^13^C-NMR (126 MHz, CDCl_3_): δ = 171.3 (C-7), 161.0 (C-1), 145.3 (C-4), 138.6 (C-2), 109.1 (C-3), 58.4 (C-5), 52.4 (C-6), 20.9 (C-8). The spectroscopic data correspond to values in the literature [43].

## Figures and Tables

**Table 1:** Scope for transformation of allylic amides **12a–d** ^a^.

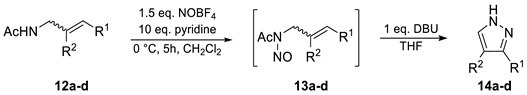				
Entry	R^1^	R^2^		14 (Yield via Two Steps %)
1	H	H	**a**	43 ^b^
2	Pr	H	**b**	23 ^b^
3	Me	H	**c**	12 ^c^
4	H	Me	**d**	5 ^c^

^a^ Reaction conditions: substrate (1.0 eq., 0.44–1.49 mmol scale), CH_2_Cl_2_ (0.6 M), THF (0.6 M) ^b^ Isolated yield, ^c^ Yield determined via quantitative NMR.

**Table 2:** Scope and limitations of method A (base) and B (thermal conditions) ^a^.

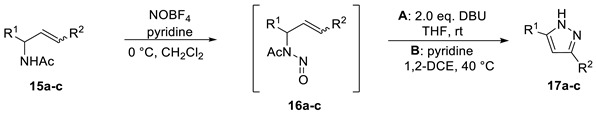					
Entry	Substrate	Configuration DB		Yield 17 (%) Method A ^d^	Yield 17 (%) Method B ^e^
1	R^1^ = H, R^2^ = CO_2_Et	*E:Z* 20:1	**a**	decomposition	14 ^b^
2	R^1^ = CH_2_OAc, R^2^ = CO_2_Et	*E*	**b**	decomposition	15 ^c^
3	R^1^ = CH_2_OAc, R^2^ = CO_2_Me	*Z*	**c**	21	63 ^c^

^a^ Reaction conditions: substrate (1.0 eq., 0.082–0.29 mmol scale), ^b^ NOBF_4_ (1.5 eq.), pyridine (10 eq.), CH_2_Cl_2_ (0.6 M), ^c^ NOBF_4_ (2 eq.), pyridine (10 eq.), CH_2_Cl_2_ (0.1 M), ^d^ THF (0.6 M), ^e^ pyridine (1.3 M), 1,2-DCE (0.1 M).

**Table 3:** Temperature and solvent screening for conversion of substrate (**15c**) to pyrazole (**17c**) ^a^.

Entry	Solvent/Base	Temperature [°C]	Reaction Time [h]	Yield (8c) [%]
1	CH_2_Cl_2_/pyridine	40	15	25
2	ClCH_2_CH_2_Cl/pyridine	40	20	63
3	CDCl_3_/pyridine	40	20	60
4	ClCH_2_CH_2_Cl/pyridine	23	20	3
5	CDCl_3_/pyridine	23	20	2
6	ClCH_2_CH_2_Cl/pyridine	60	20	11
7	CDCl_3_/pyridine	60	20	16

^a^ Reaction conditions: substrate (1.0 equiv., 0.10–0.11 mmol scale), pyridine (1.2 M), solvent (0.1 M), 20 h.

## Data Availability

Contained in supporting information.

## Supplementary Materials

The following supporting information can be downloaded at: https://www.mdpi.com/article/10.3390/molecules29204885/s1, Figures S1–S40: 1H- and 13C-NMR spectra.
