# Supplementary material for: Serendipitous Conversion of an Acetylamino Dideoxy-Octonic Acid Derivate into a Functionalized Carbohydrate–Pyrazole Conjugate and Investigation of the Method´s General Applicability
Source: Molecules. 2024 Oct 15;29(20):4885. doi: 10.3390/molecules29204885 (PMC11509885; doi:10.3390/molecules29204885)
Supplement: Supplementary file 1 [file molecules-29-04885-s001.zip › molecules-3206990-supplementary.pdf]

Supporting Information

Serendipitous conversion of an ADOA derivative into functionalized pyrazole and investigation of the method's general applicability

Jelena K. Berl, Christian Czaschke, Ann-Kathrin Pramor, Christian B. W. Stark, * and Joachim Thiem *

Department of Chemistry, Institute for Organic Chemistry, University of Hamburg, Martin-Luther-King-Platz 6, D-20146 Hamburg, Germany

* Emails: christian.stark@uni-hamburg.de; joachim.thiem@uni-hamburg.de

^{1}H- and ^{13}C-NMR spectra S1-40

^{1}H- and ^{13}C-NMR spectra

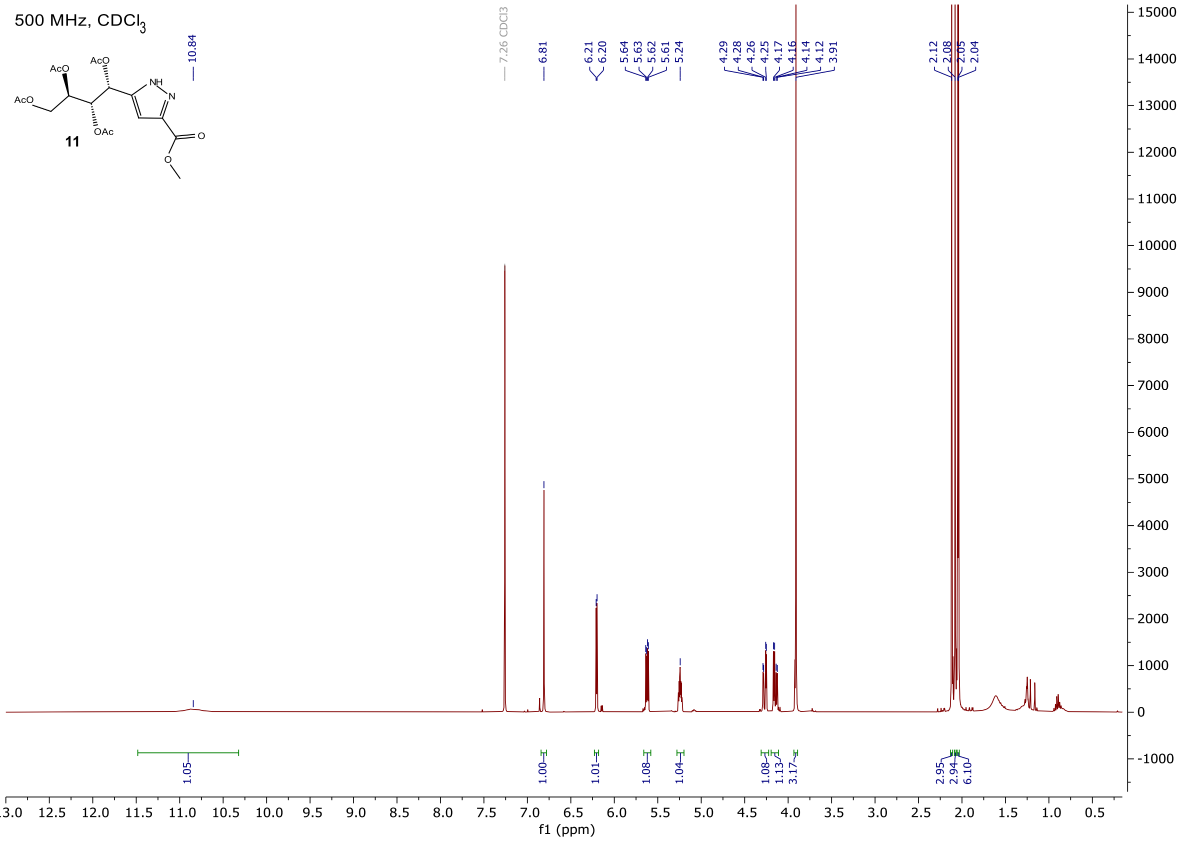

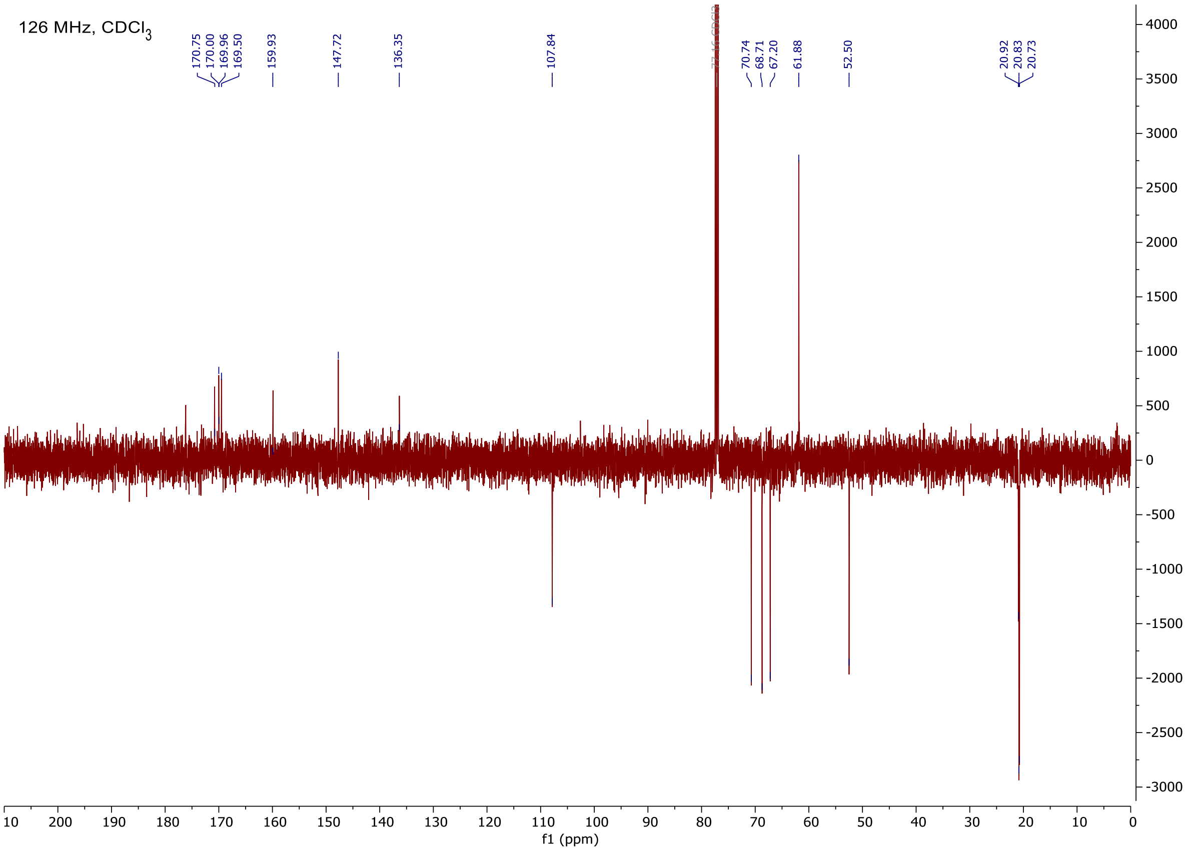
126 MHz, CDCl3
170.75
170.00
169.96
169.50
159.93
147.72
136.35
107.84
70.74
68.71
67.20
61.88
52.50
20.92
20.83
20.73
f1 (ppm)

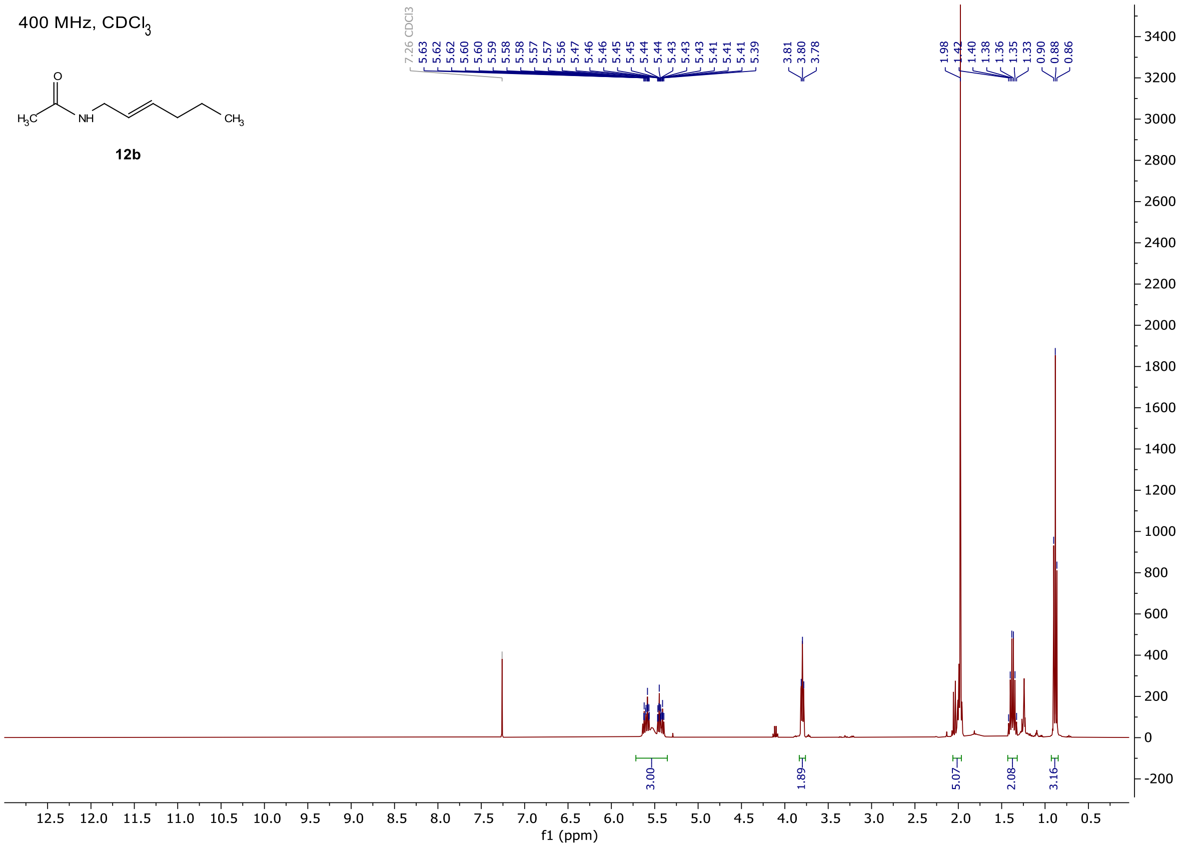
400 MHz, CDCl3
O
H3C
NH
CH3
12b
7.26 CDCl3
5.63
5.62
5.62
5.60
5.59
5.58
5.58
5.57
5.57
5.56
5.47
5.46
5.45
5.45
5.44
5.44
5.43
5.43
5.43
5.41
5.41
5.41
5.39
3.81
3.80
3.78
1.98
1.42
1.40
1.38
1.36
1.35
1.33
0.90
0.88
0.86
3.00
1.89
5.07
2.08
3.16
f1 (ppm)

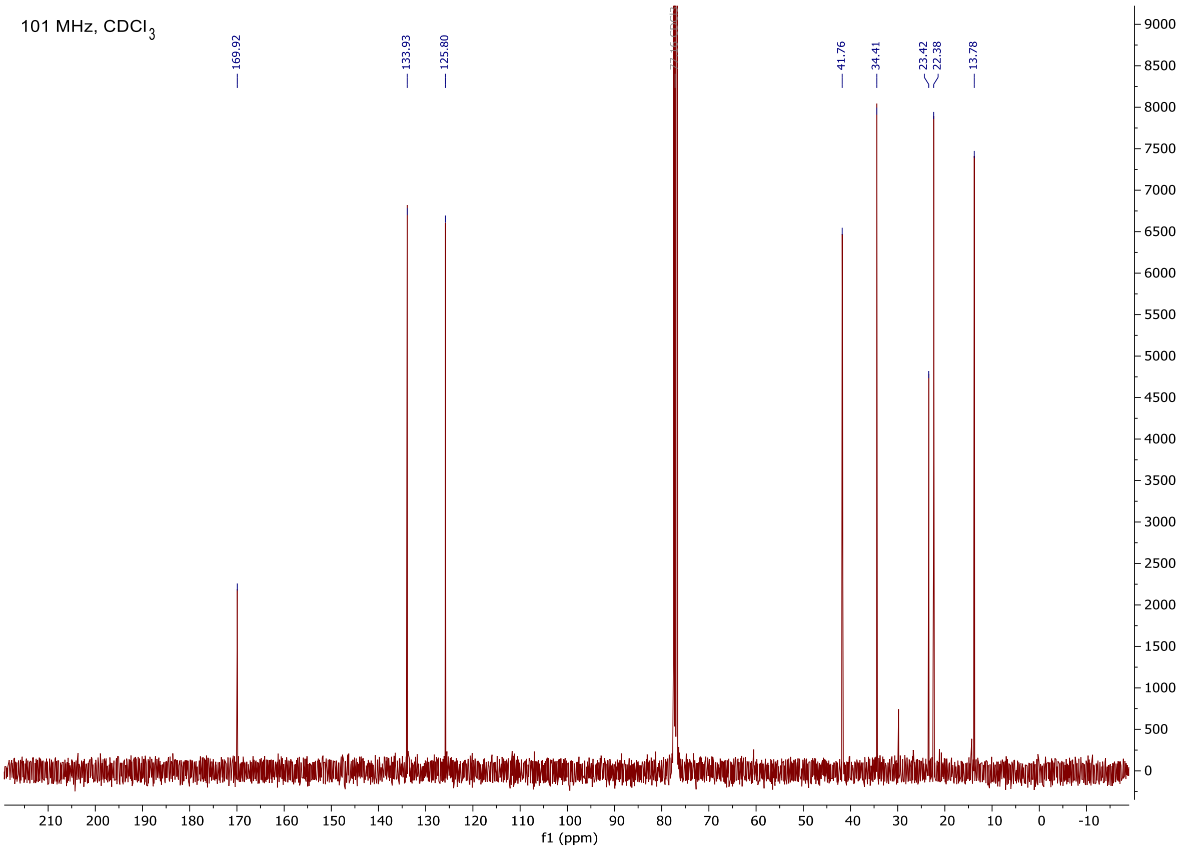

101 MHz, $CDCl_3$
169.92
133.93
125.80
41.76
34.41
23.42
22.38
13.78
f1 (ppm)

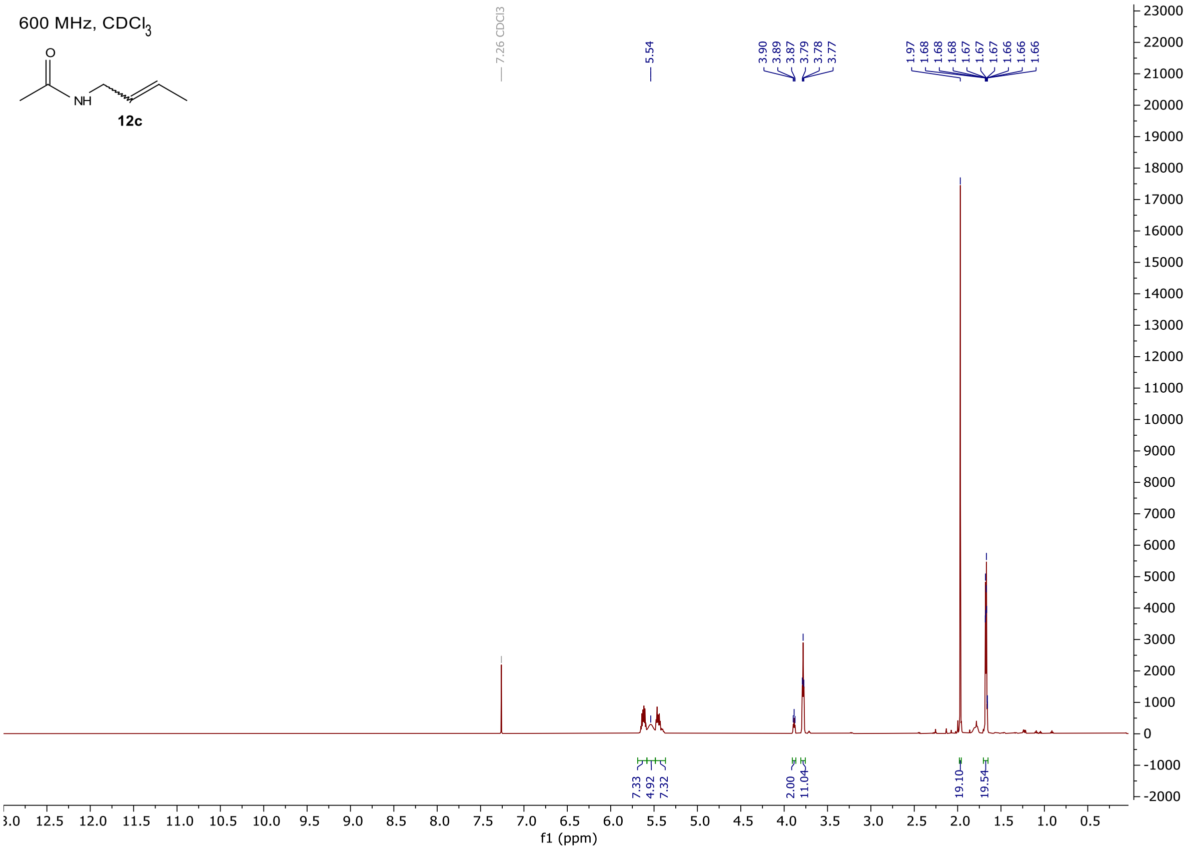

600 MHz, $CDCl_3$
O
NH
12c
7.26 CDCl3
5.54
3.90
3.89
3.87
3.79
3.78
3.77
1.97
1.68
1.68
1.68
1.67
1.67
1.67
1.66
1.66
1.66
7.33
4.92
7.32
2.00
11.04
19.10
19.54
f1 (ppm)

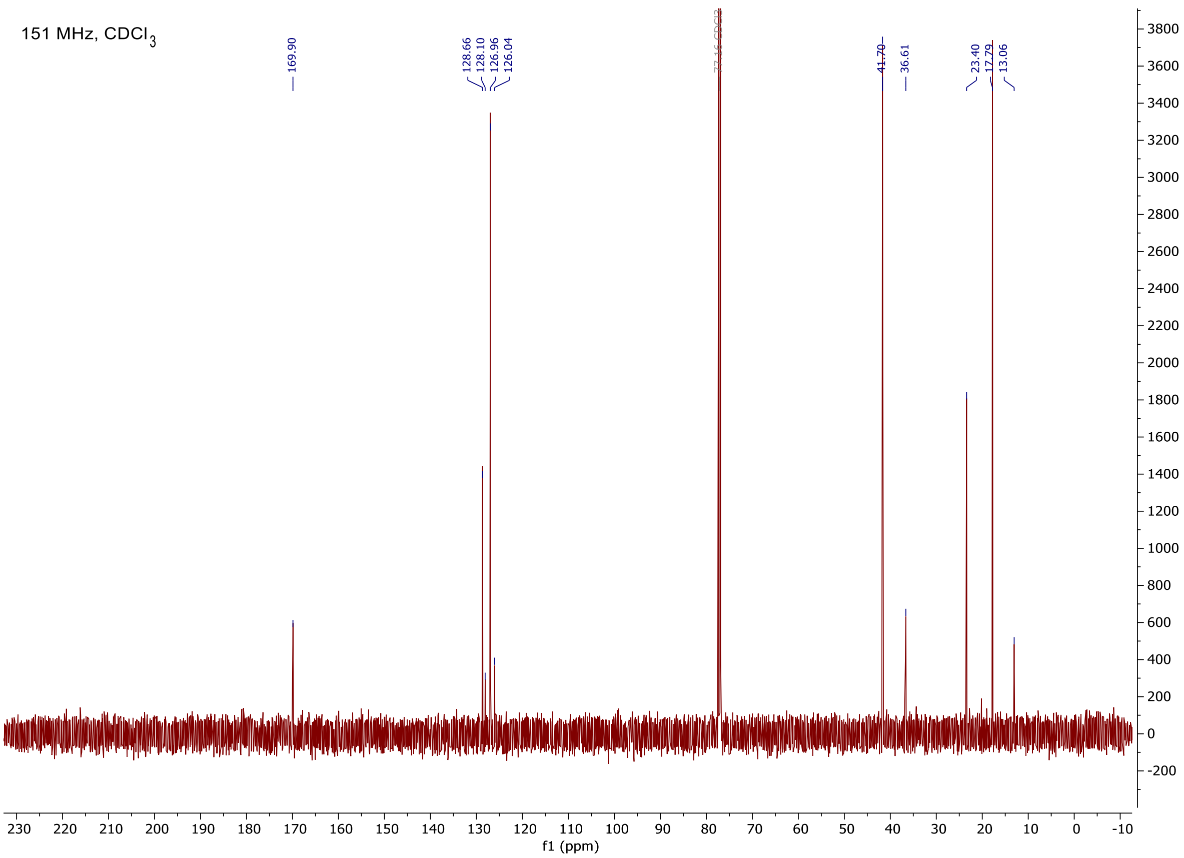
151 MHz, CDCl3
169.90
128.66
128.10
126.96
126.04
36.61
23.40
13.06
f1 (ppm)

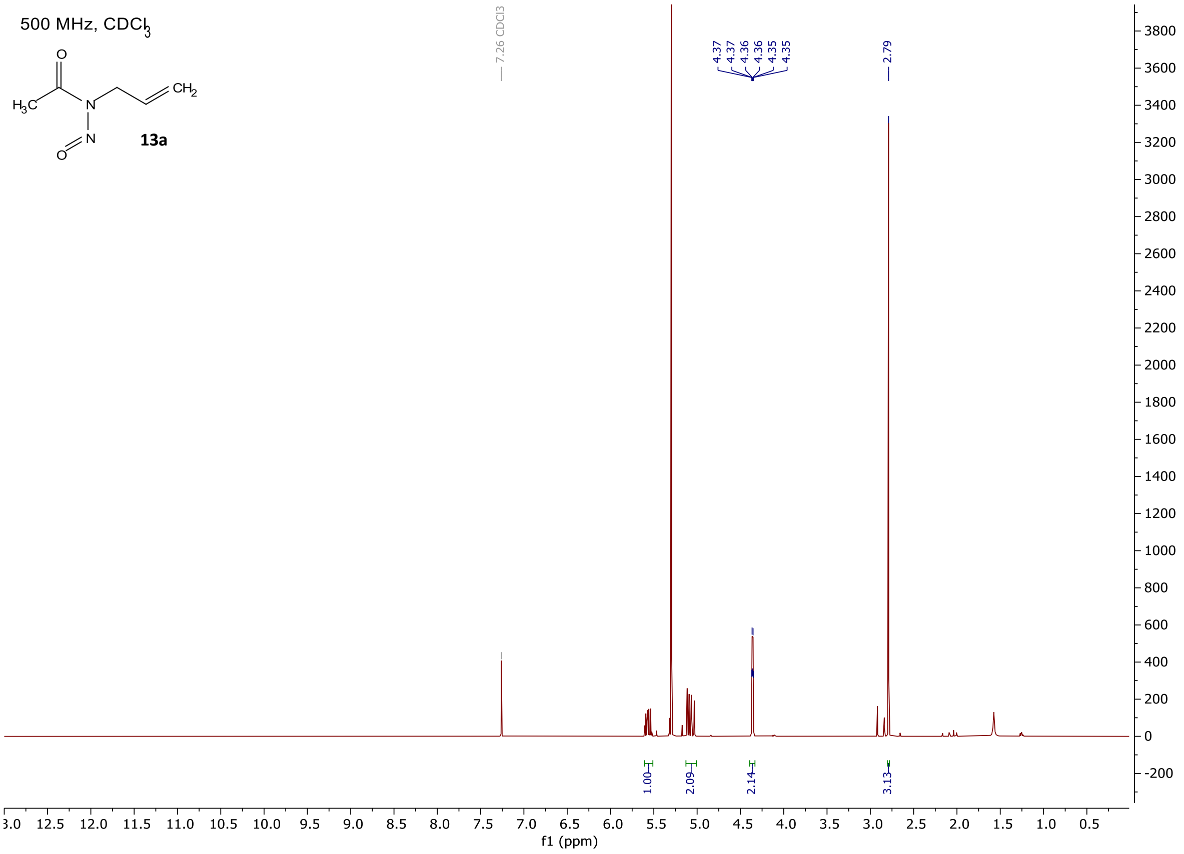
500 MHz, CDCl3
O
H3C
N
CH2
N
O
13a
7.26 CDCl3
4.37
4.37
4.36
4.36
4.35
4.35
2.79
1.00
2.09
2.14
3.13
f1 (ppm)

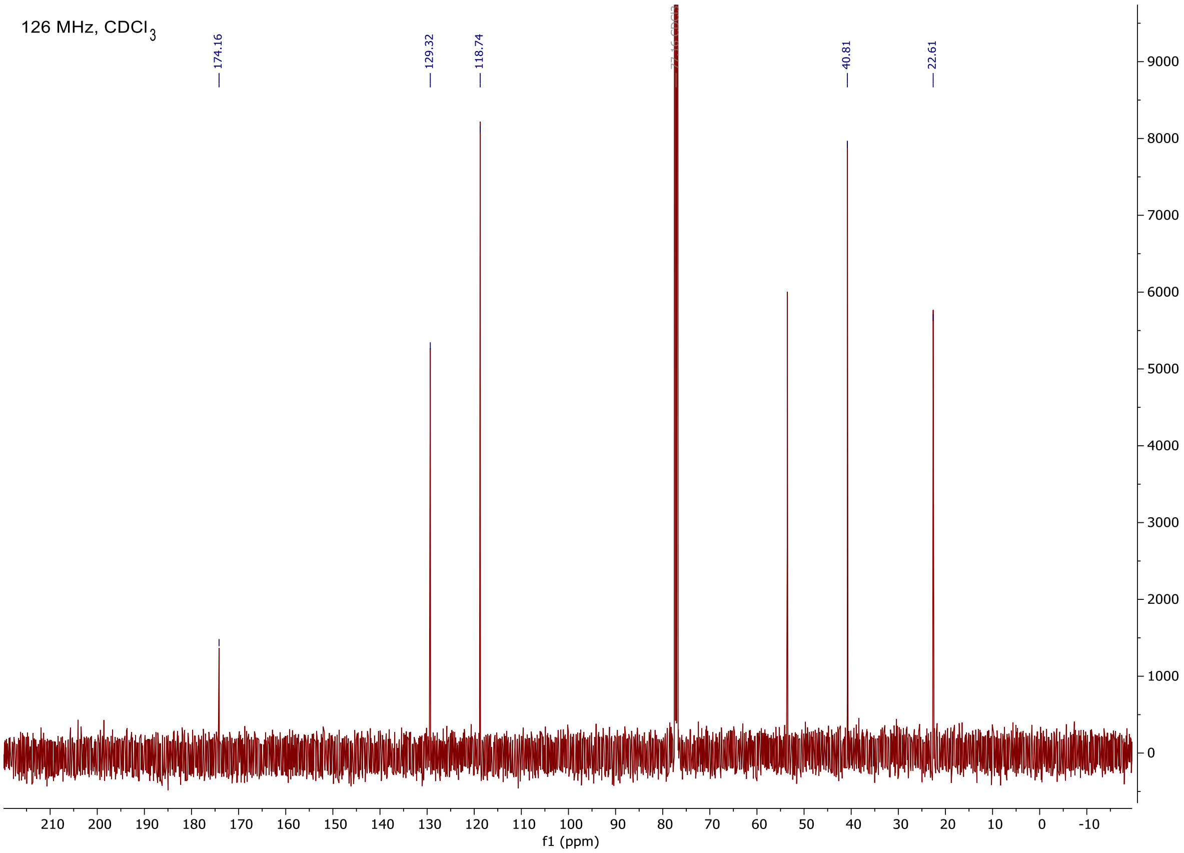
126 MHz, CDCl3
174.16
129.32
118.74
77.16 CDCl3
40.81
22.61
f1 (ppm)

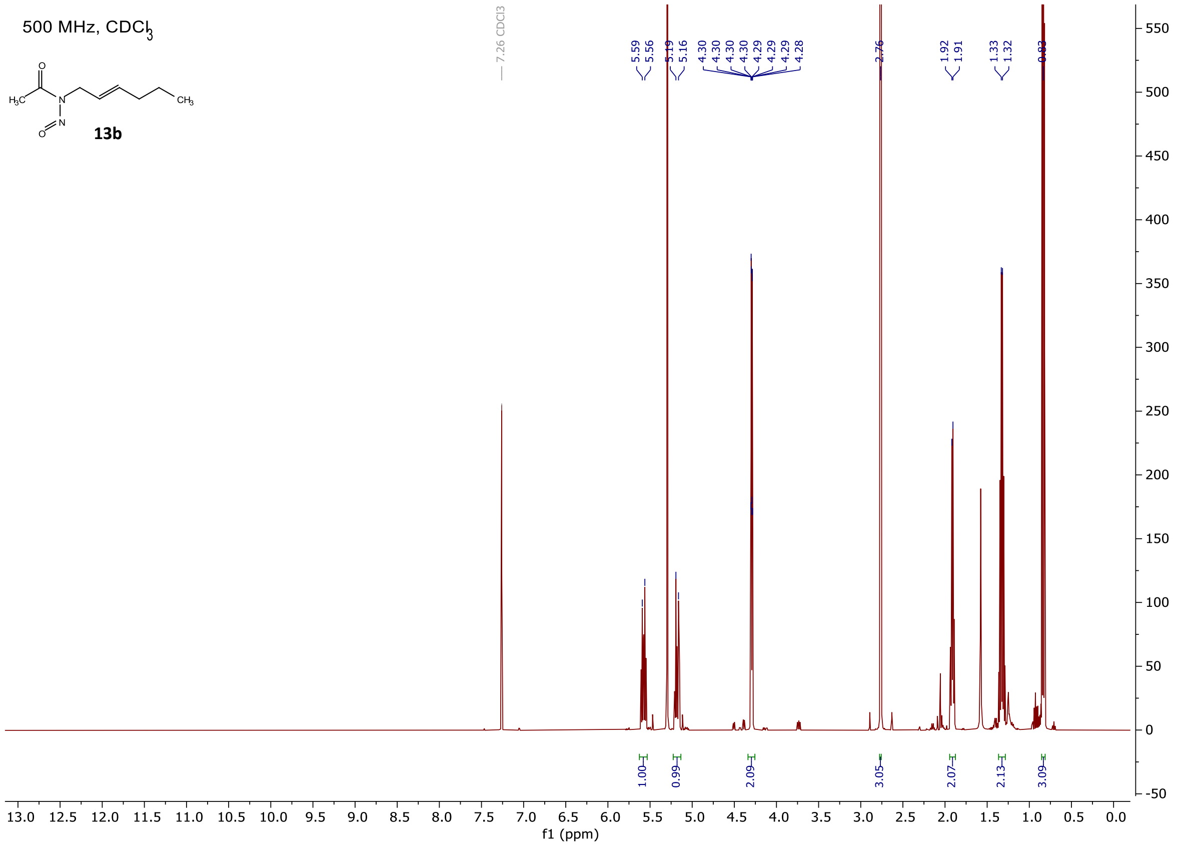
500 MHz, CDCl3
7.26 CDCl3
5.59
5.56
5.19
5.16
4.30
4.30
4.30
4.30
4.29
4.29
4.29
4.28
2.76
1.92
1.91
1.33
1.32
0.83
1.00
0.99
2.09
3.05
2.07
2.13
3.09
f1 (ppm)

13b

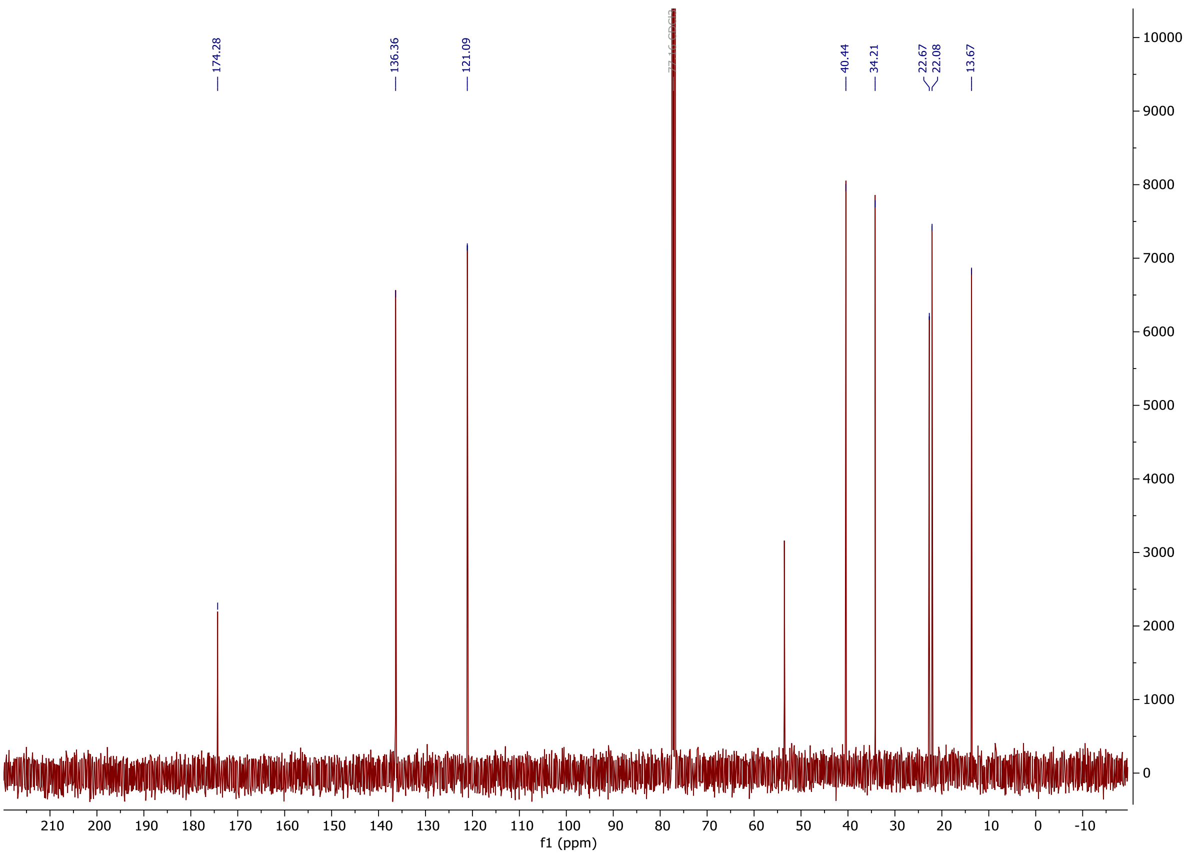
174.28
136.36
121.09
77.16 CDCl3
40.44
34.21
22.67
22.08
13.67
f1 (ppm)

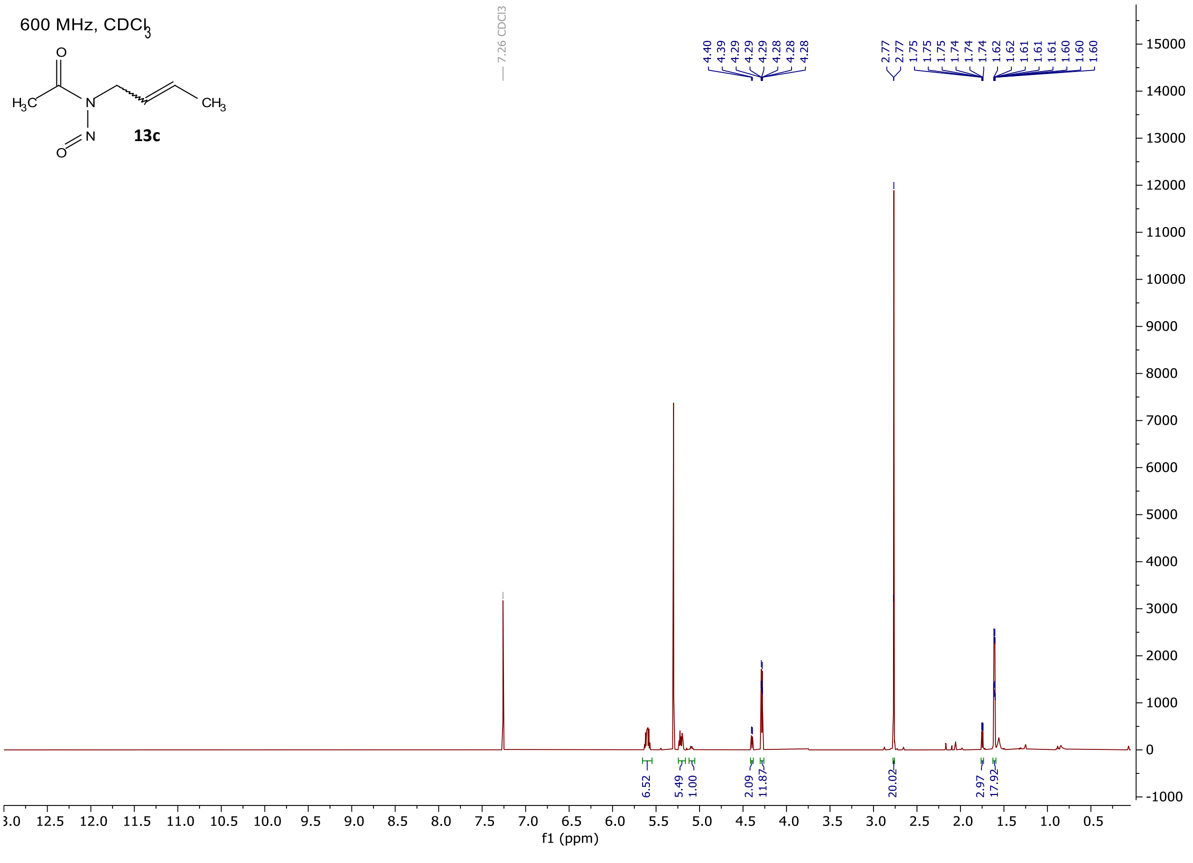
600 MHz, CDCl3
13c
7.26 CDCl3
f1 (ppm)

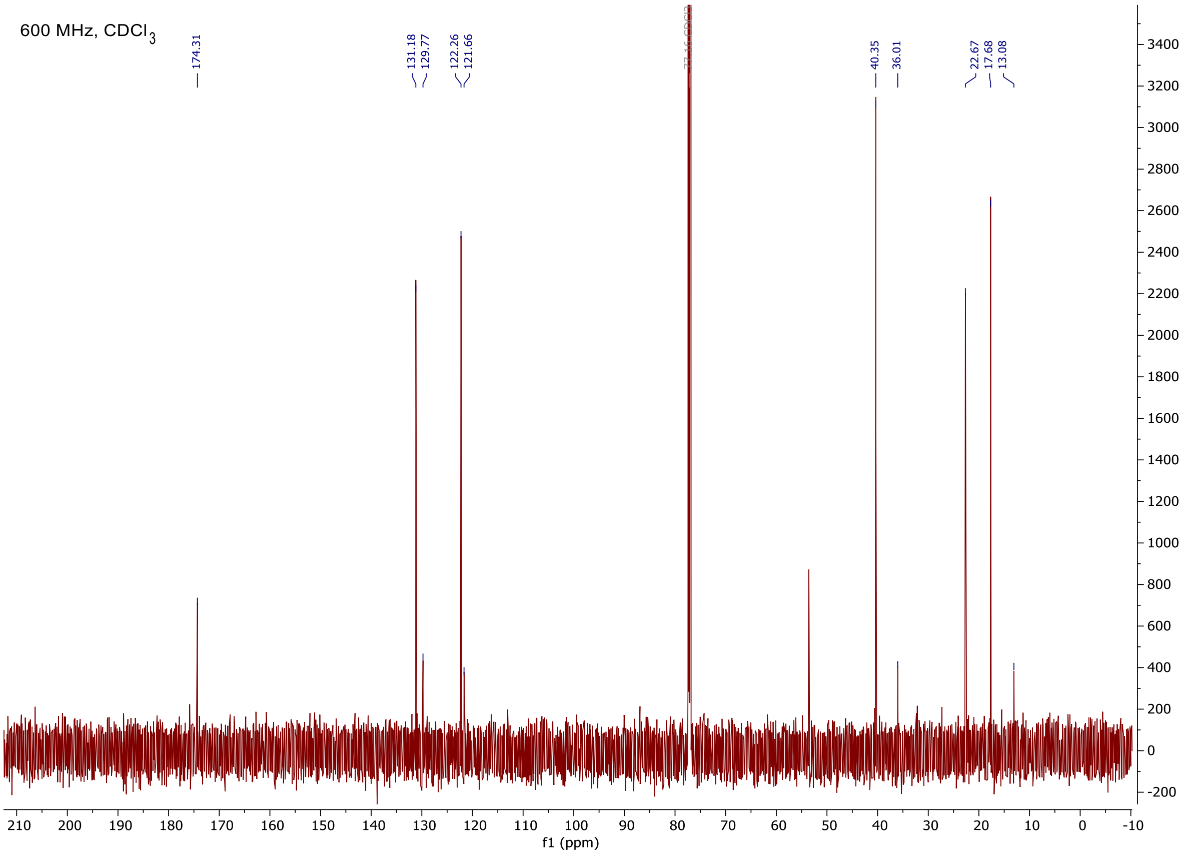
600 MHz, CDCl3
174.31
131.18
129.77
122.26
121.66
40.35
36.01
22.67
17.68
13.08
f1 (ppm)

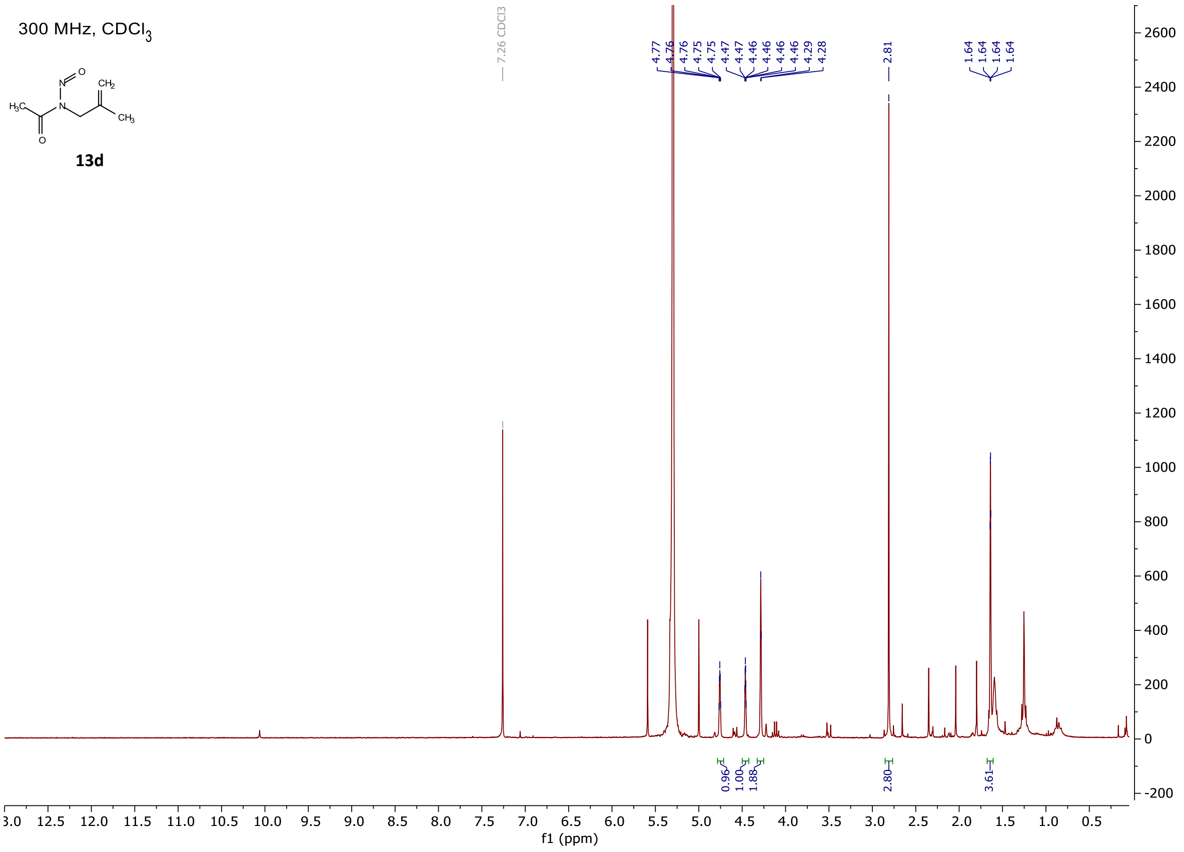
300 MHz, CDCl3
13d
7.26 CDCl3
4.77
4.76
4.76
4.75
4.75
4.47
4.47
4.46
4.46
4.46
4.46
4.29
4.28
2.81
1.64
1.64
1.64
1.64
0.96
1.00
1.88
2.80
3.61
f1 (ppm)

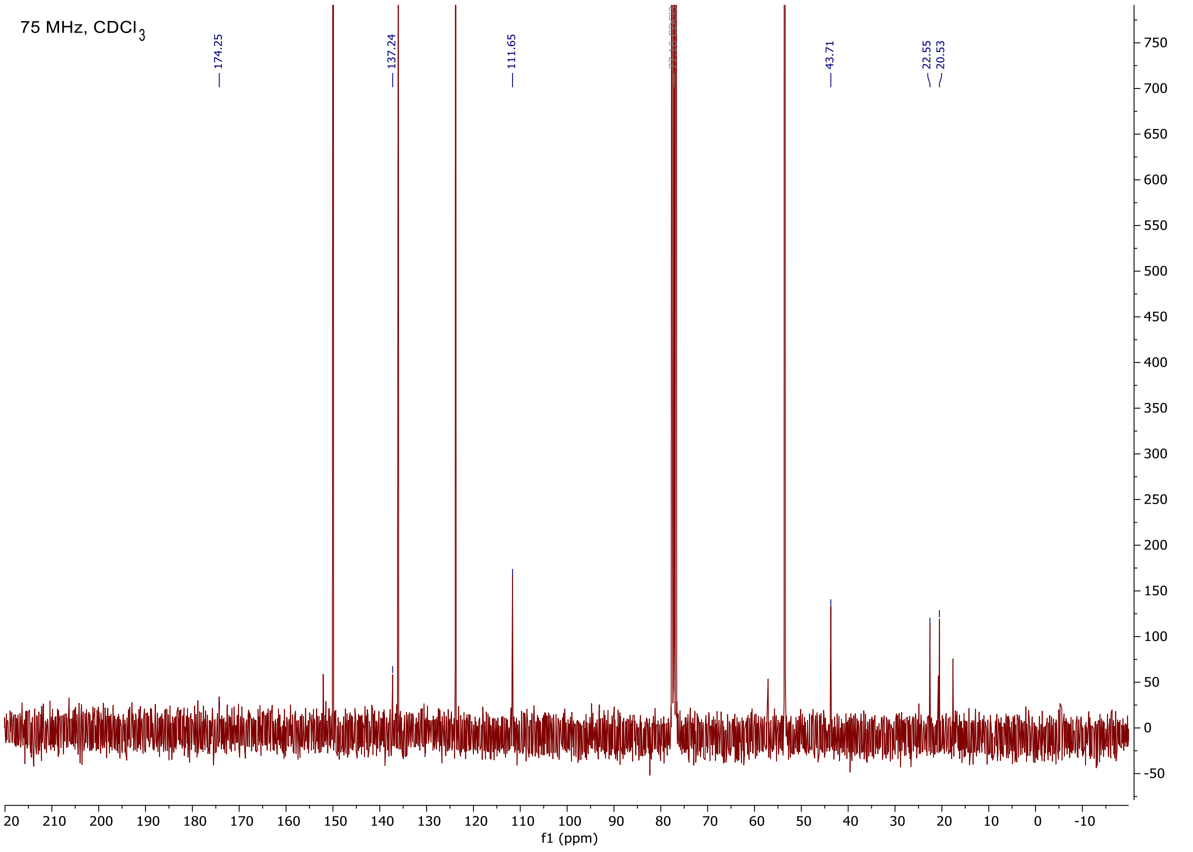
75 MHz, CDCl3
174.25
137.24
111.65
43.71
22.55
20.53
f1 (ppm)

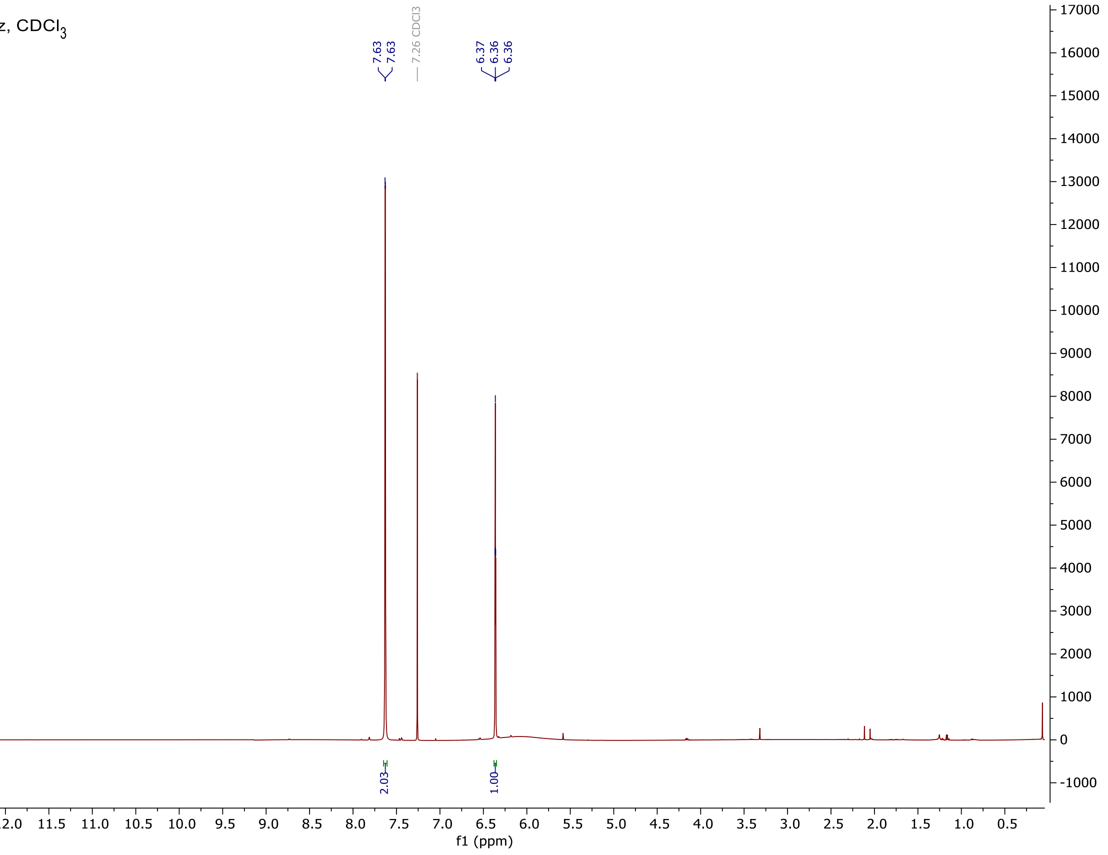
500 MHz, CDCl3
7.63
7.63
7.26 CDCl3
6.37
6.36
6.36
2.03
1.00
f1 (ppm)

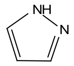
NH
N

14a

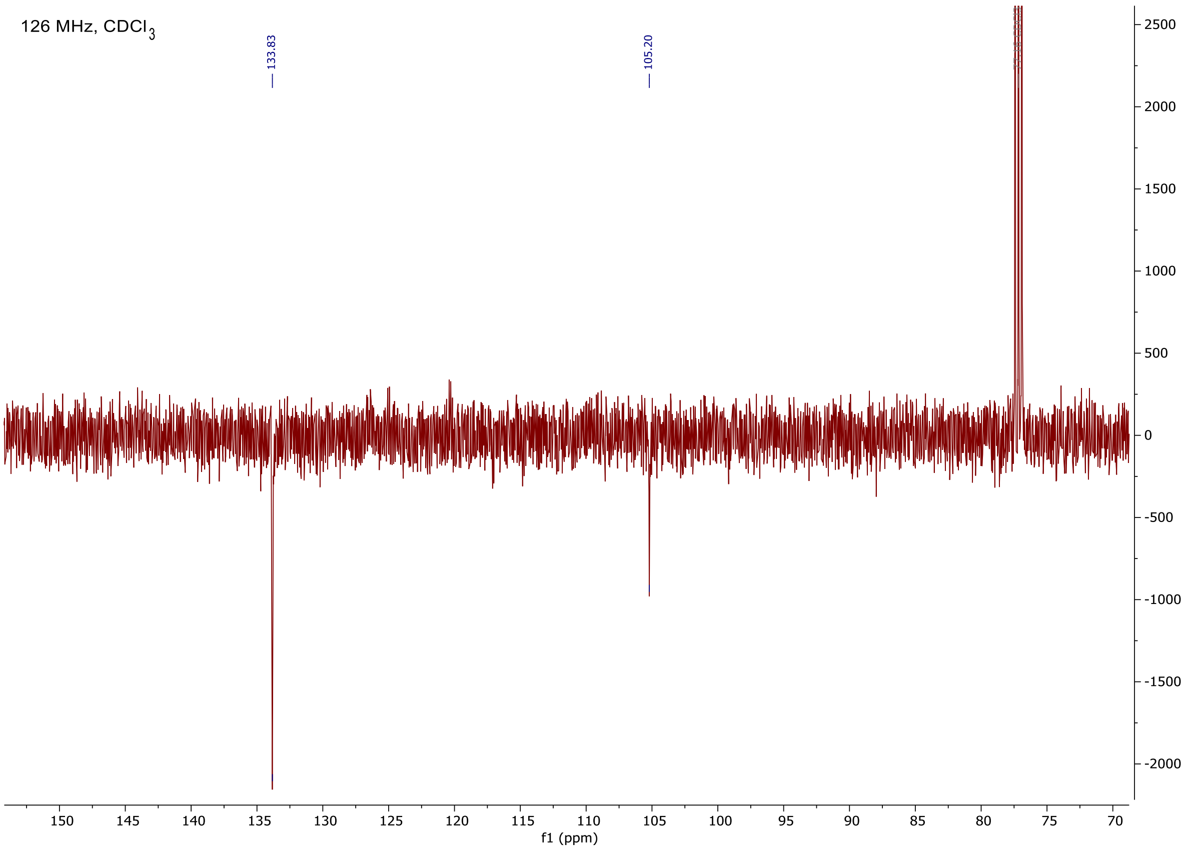
126 MHz, CDCl3
133.83
105.20
f1 (ppm)

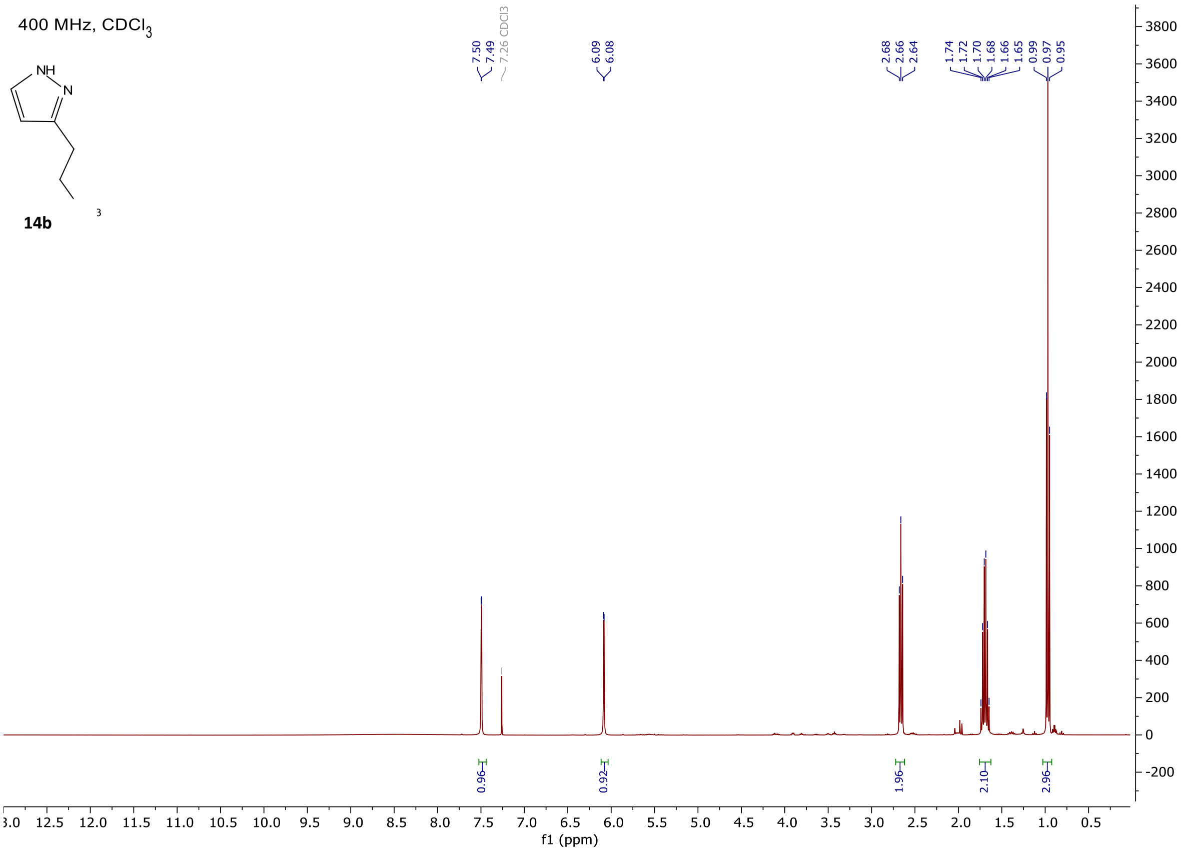
400 MHz, CDCl3
NH
N
7.50
7.49
7.26 CDCl3
6.09
6.08
2.68
2.66
2.64
1.74
1.72
1.70
1.68
1.66
1.65
0.99
0.97
0.95
0.96
0.92
1.96
2.10
2.96
f1 (ppm)

14b

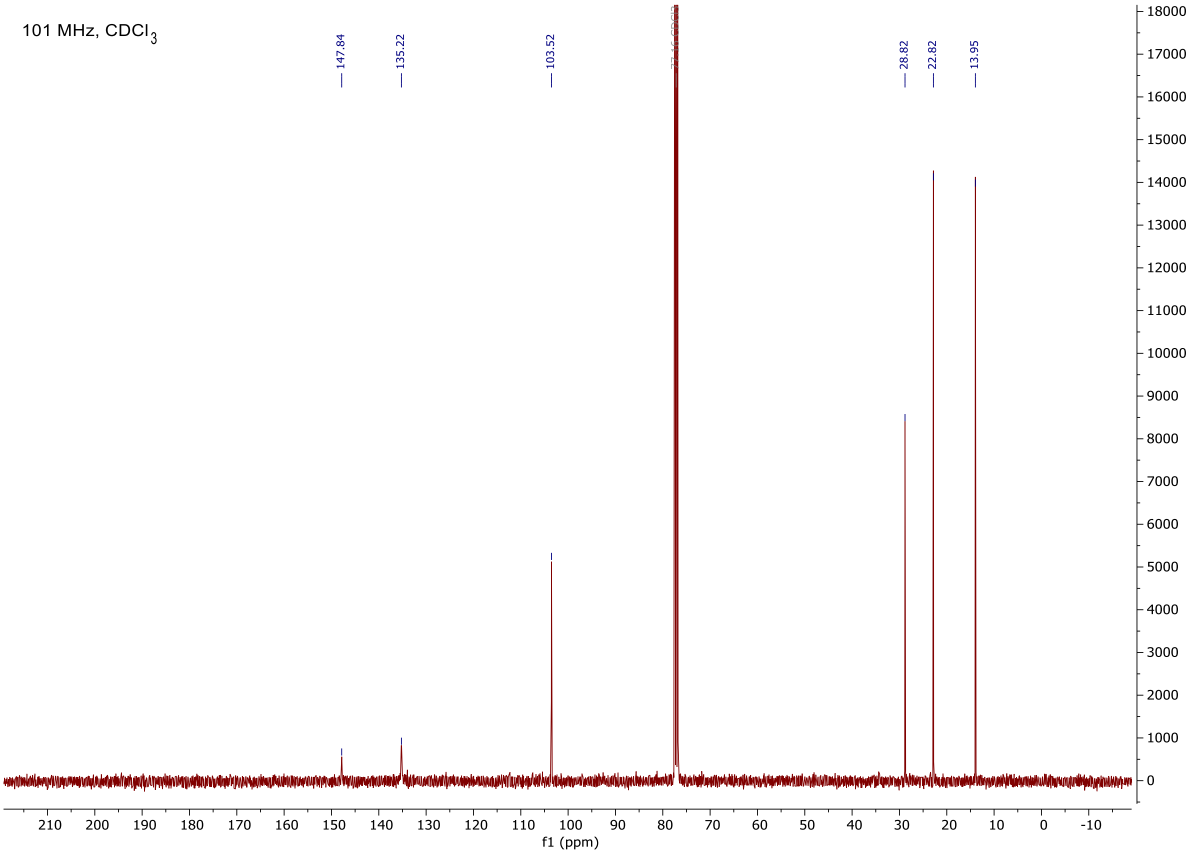
101 MHz, CDCl3
147.84
135.22
103.52
28.82
22.82
13.95
f1 (ppm)

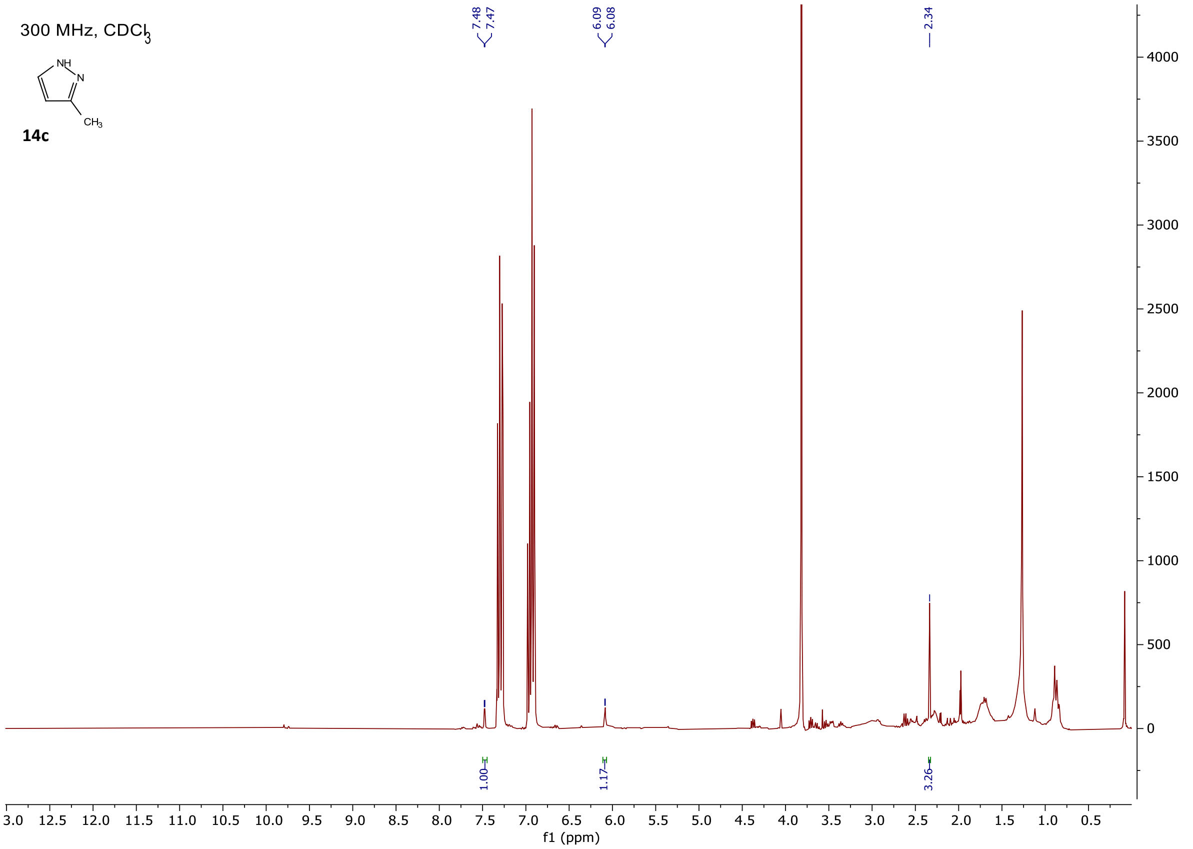
300 MHz, CDCl3
NH
N
CH3
14c
7.48
7.47
6.09
6.08
2.34
1.00
1.17
3.26
f1 (ppm)

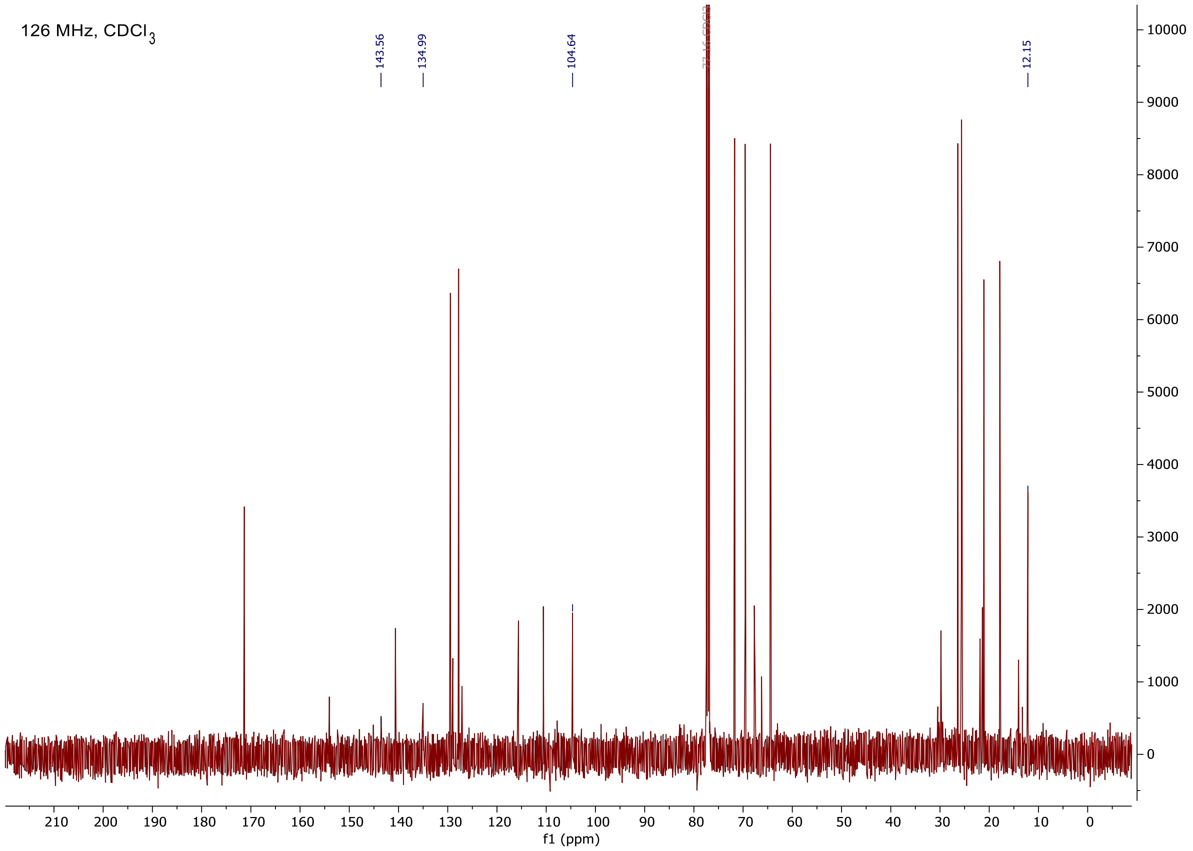
126 MHz, CDCl3
143.56
134.99
104.64
12.15
f1 (ppm)

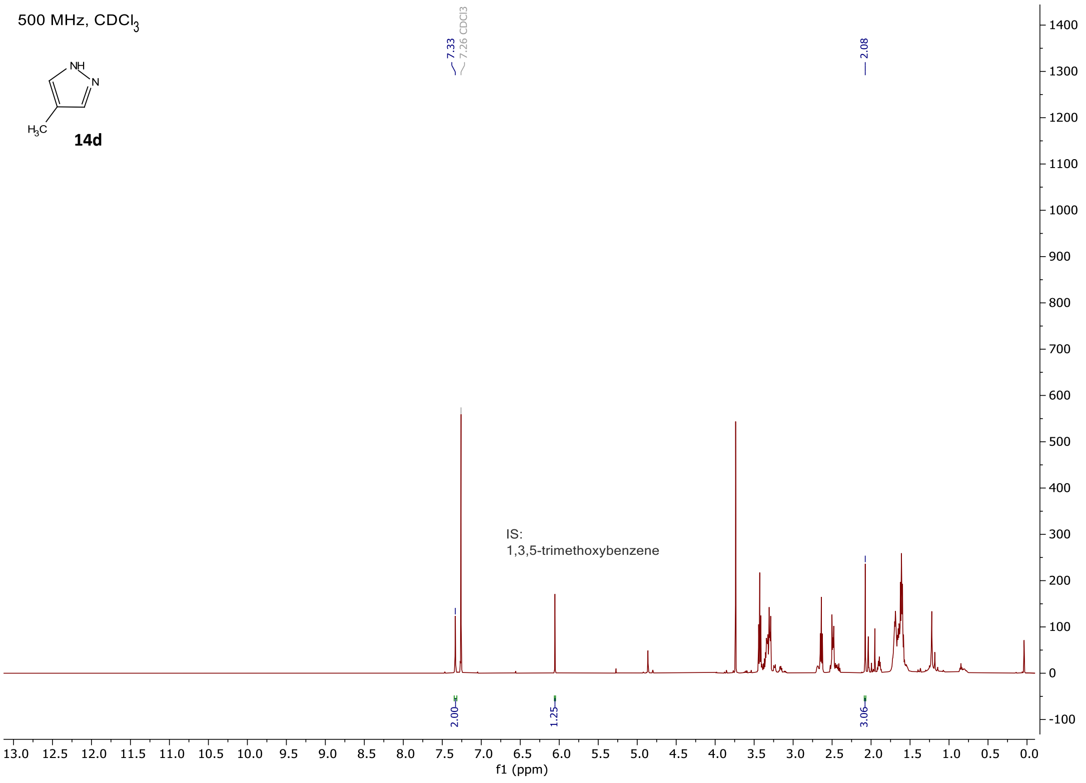
500 MHz, CDCl3
7.33
7.26 CDCl3
2.08
14d
IS:
1,3,5-trimethoxybenzene
2.00
1.25
3.06
f1 (ppm)

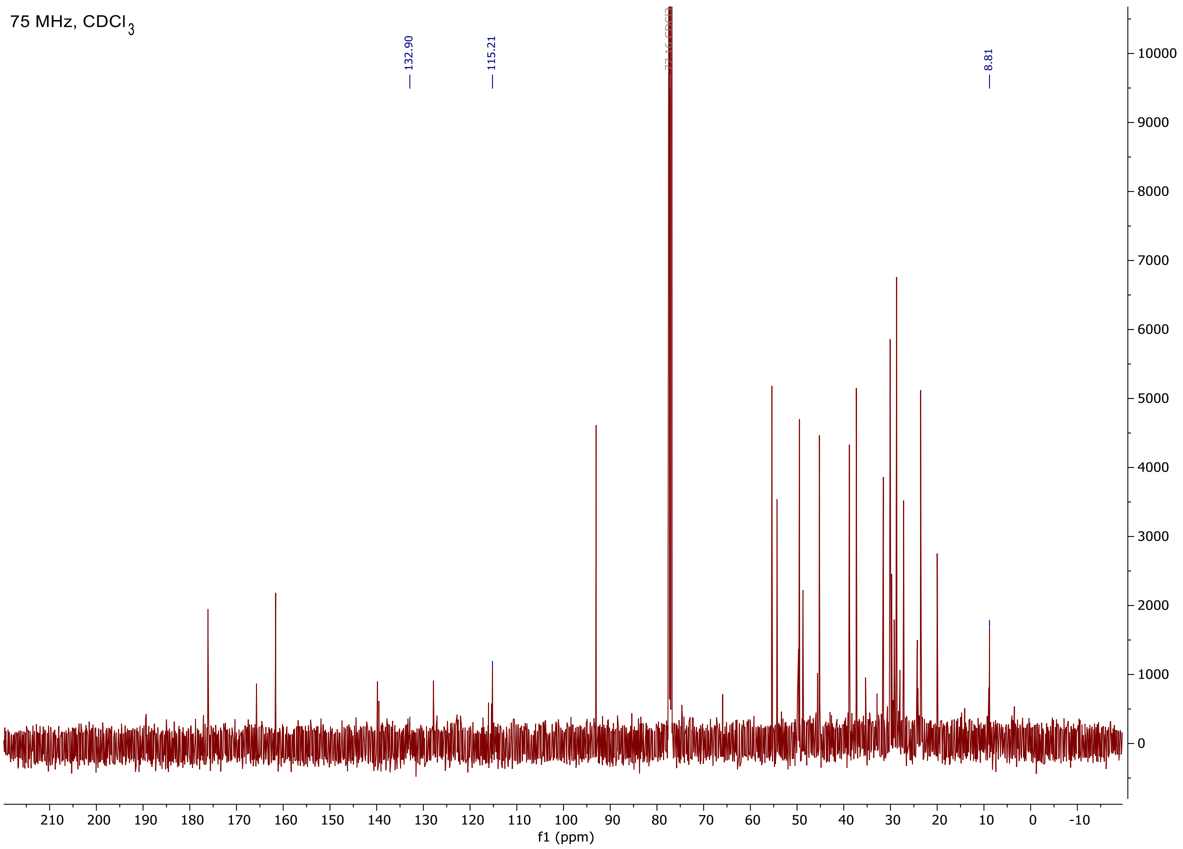

75 MHz, CDCl$_3$
132.90
115.21
8.81
f1 (ppm)

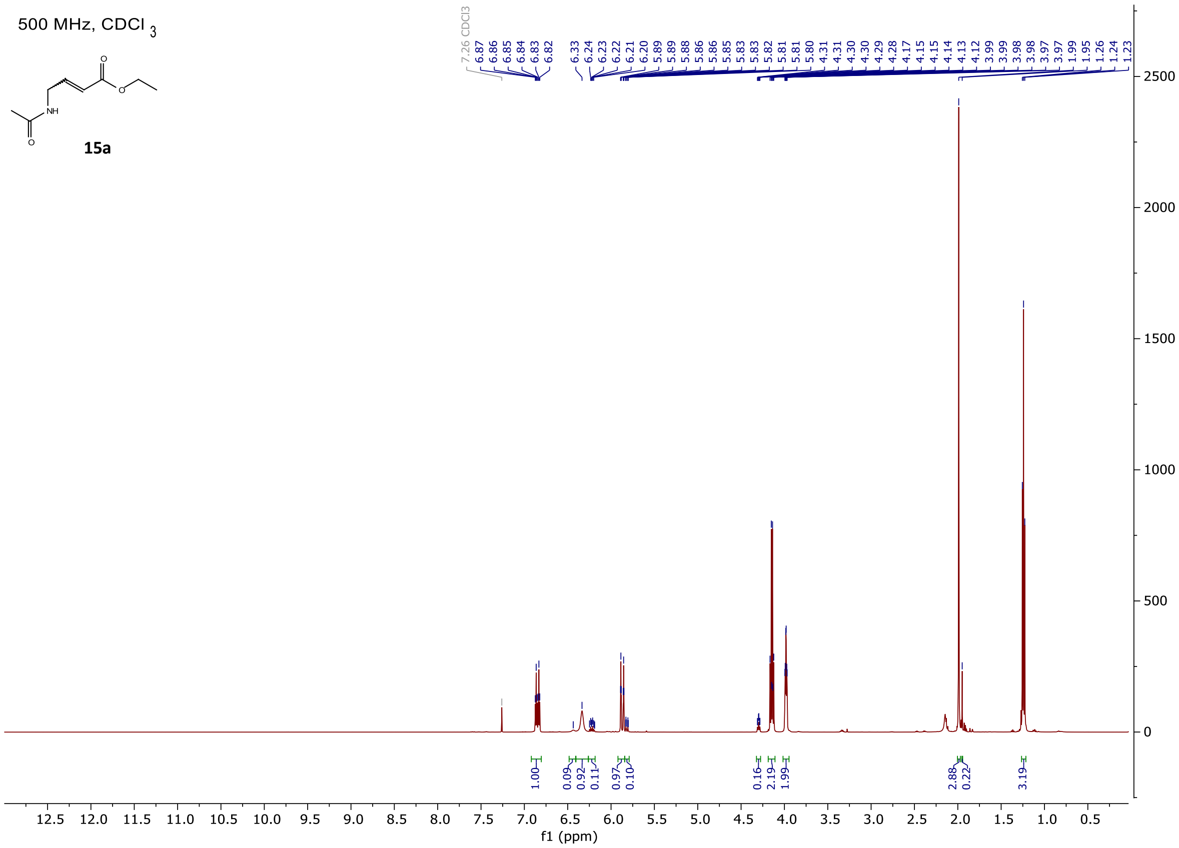

500 MHz, CDCl $_3$
7.26 CDCl3
6.87
6.86
6.85
6.84
6.83
6.82
6.33
6.24
6.23
6.22
6.21
6.20
5.89
5.88
5.86
5.85
5.83
5.83
5.82
5.81
5.80
4.31
4.31
4.30
4.29
4.28
4.17
4.15
4.15
4.14
4.13
4.12
3.99
3.99
3.98
3.98
3.97
1.99
1.95
1.26
1.24
1.23
1.00
0.09
0.92
0.11
0.97
0.10
0.16
2.19
1.99
2.88
0.22
3.19
f1 (ppm)

15a

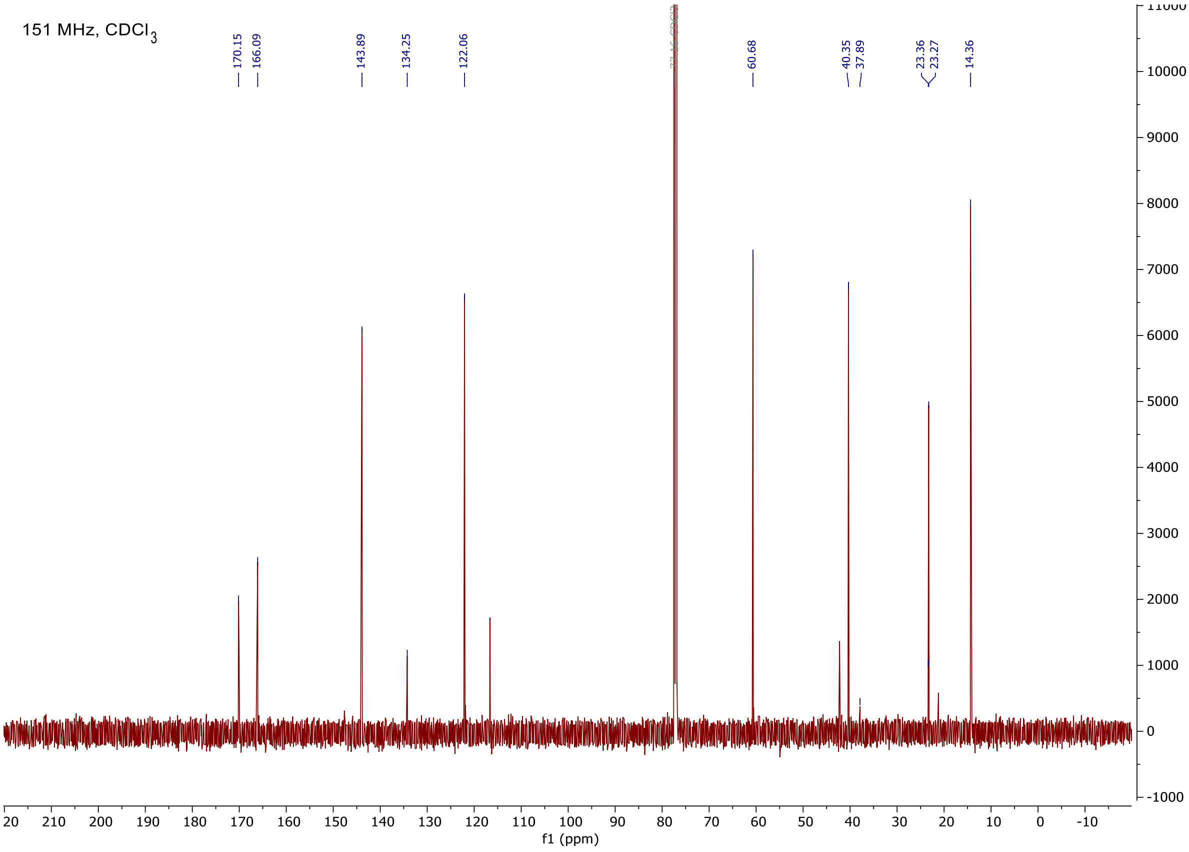
151 MHz, CDCl3
170.15
166.09
143.89
134.25
122.06
60.68
40.35
37.89
23.36
23.27
14.36
f1 (ppm)

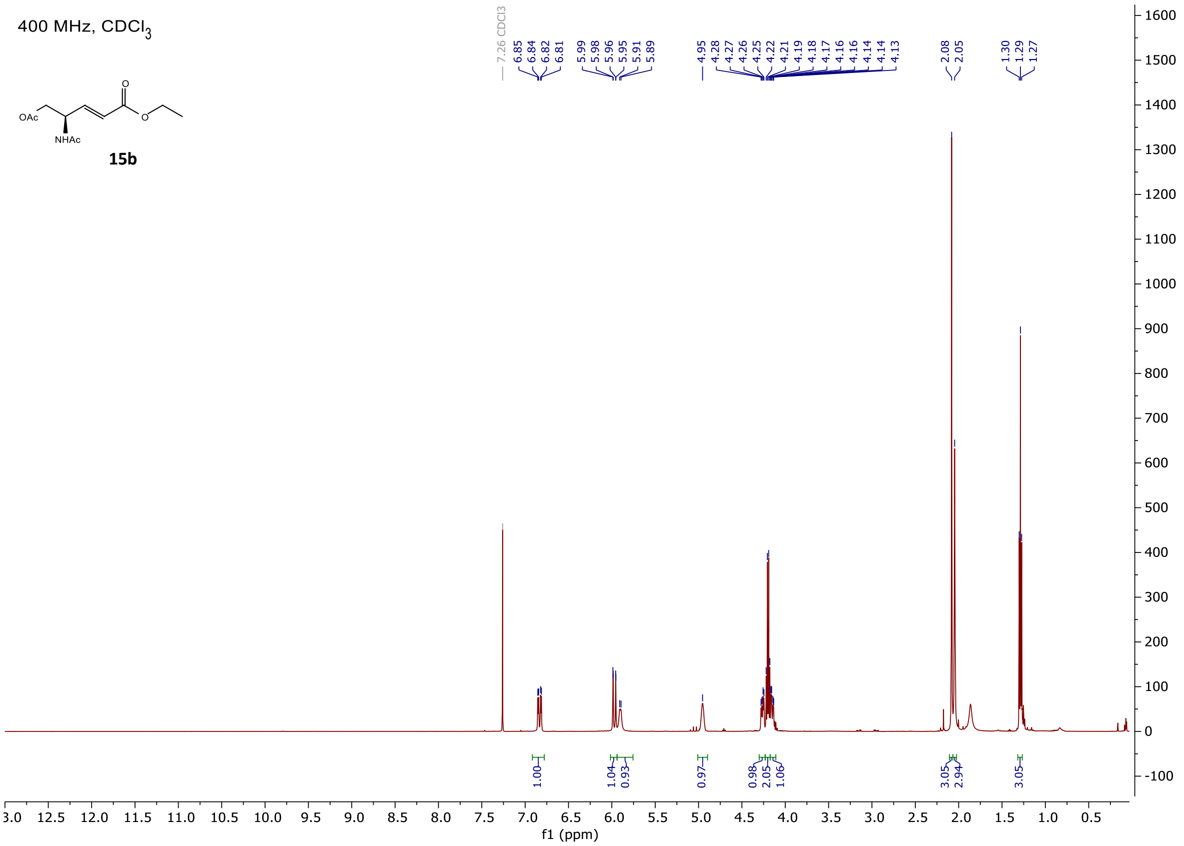
400 MHz, CDCl3
7.26 CDCl3
6.85
6.84
6.82
6.81
5.99
5.98
5.96
5.95
5.91
5.89
4.95
4.28
4.27
4.26
4.25
4.22
4.21
4.19
4.18
4.17
4.16
4.16
4.14
4.14
4.13
2.08
2.05
1.30
1.29
1.27
1.00
1.04
0.93
0.97
0.98
2.05
1.06
3.05
2.94
3.05
f1 (ppm)
OAc
NHAc
O
O

15b

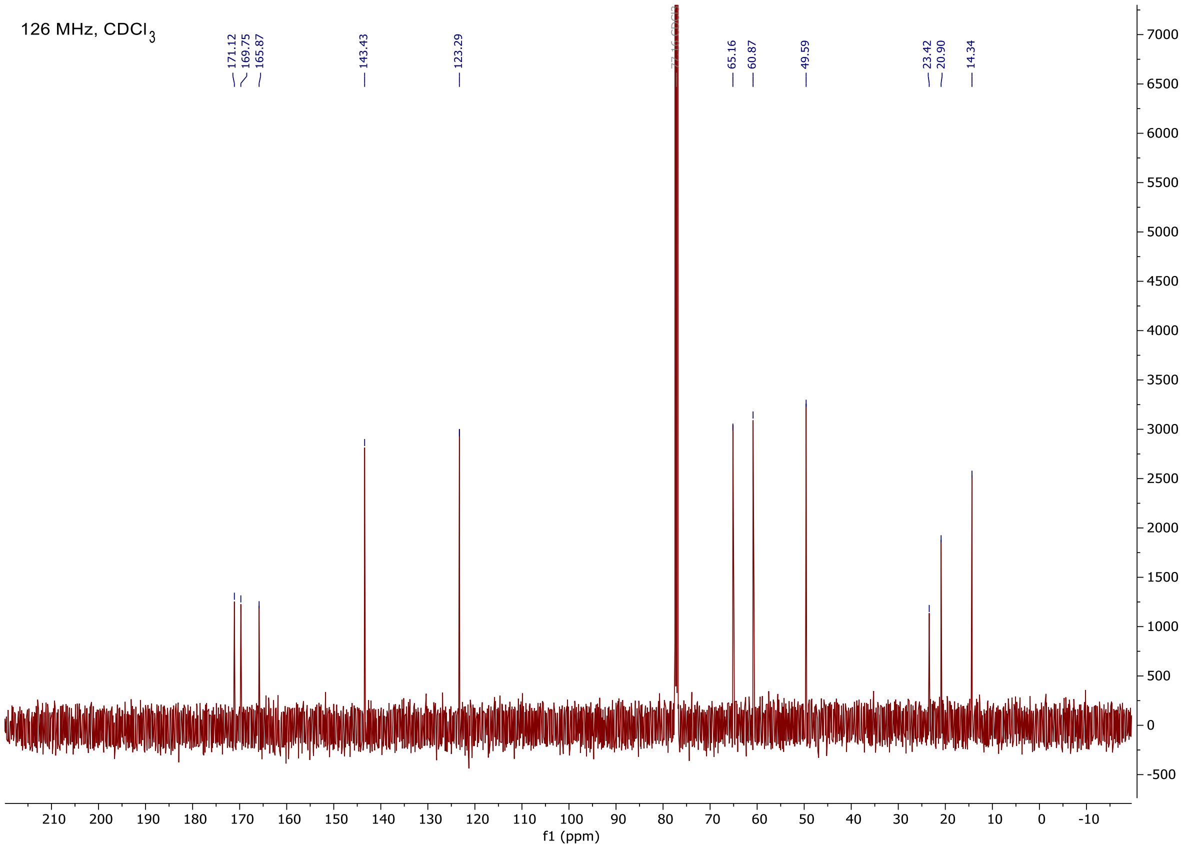

126 MHz, CDCl3
171.12
169.75
165.87
143.43
123.29
65.16
60.87
49.59
23.42
20.90
14.34
f1 (ppm)

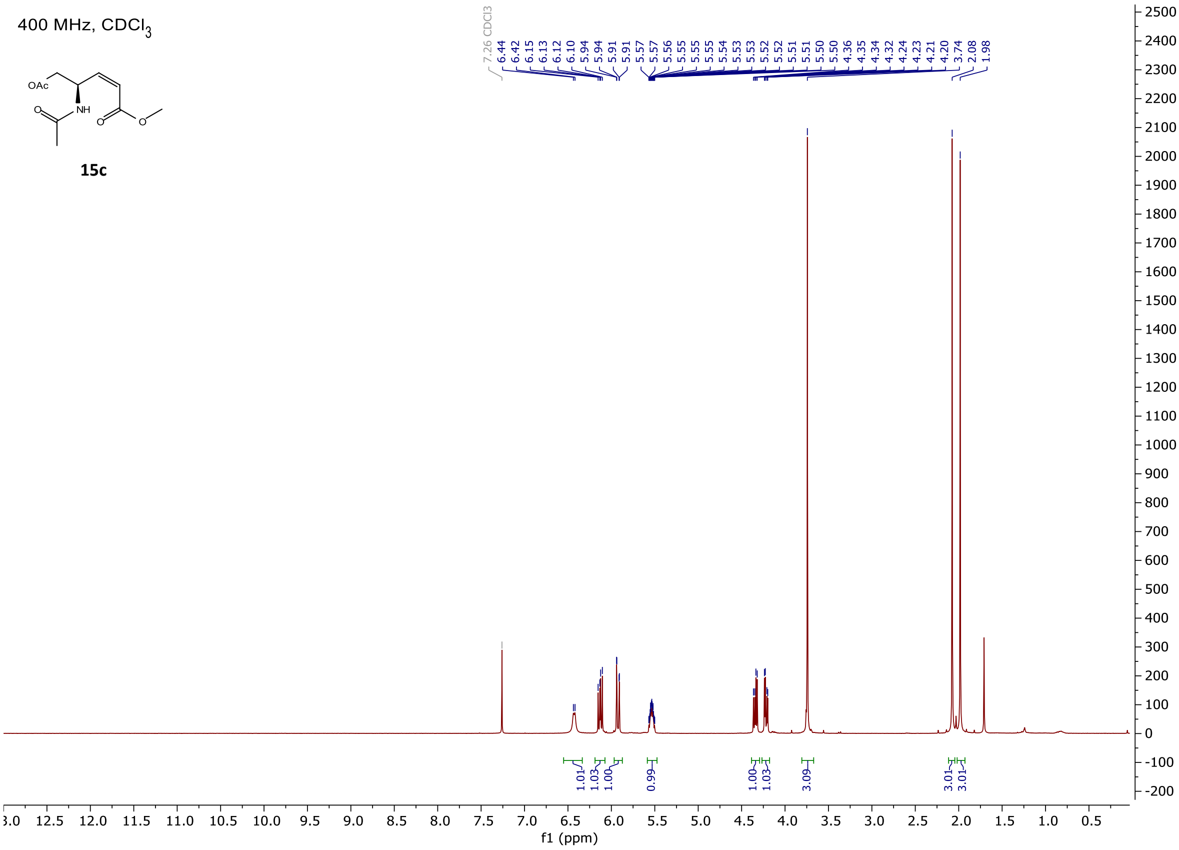

400 MHz, CDCl3
OAc
NH
15c
7.26 CDCl3
6.44
6.42
6.15
6.13
6.12
6.10
5.94
5.94
5.91
5.91
5.57
5.57
5.56
5.55
5.55
5.54
5.53
5.53
5.52
5.52
5.51
5.51
5.50
5.50
4.36
4.35
4.34
4.32
4.24
4.23
4.21
4.20
3.74
2.08
1.98
1.01
1.03
1.00
0.99
1.00
1.03
3.09
3.01
3.01
f1 (ppm)

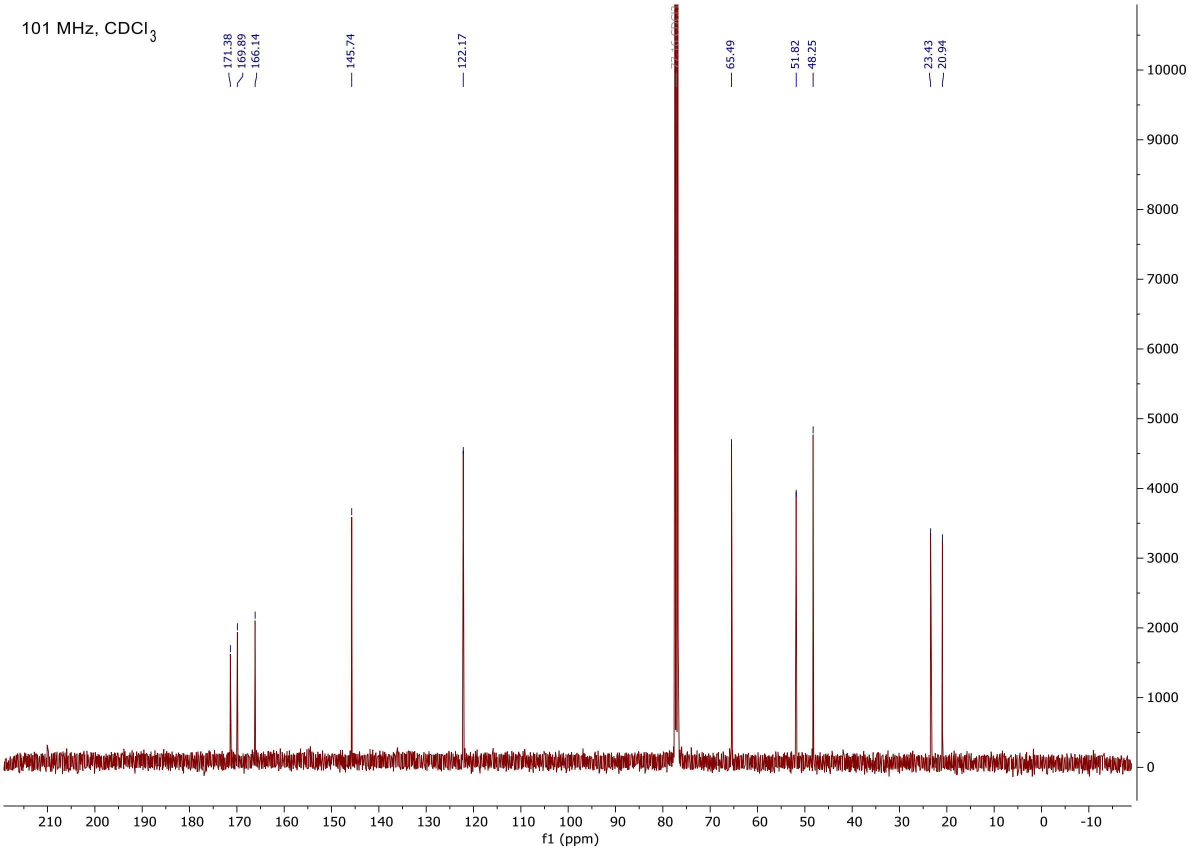
101 MHz, CDCl3
171.38
169.89
166.14
145.74
122.17
65.49
51.82
48.25
23.43
20.94
f1 (ppm)

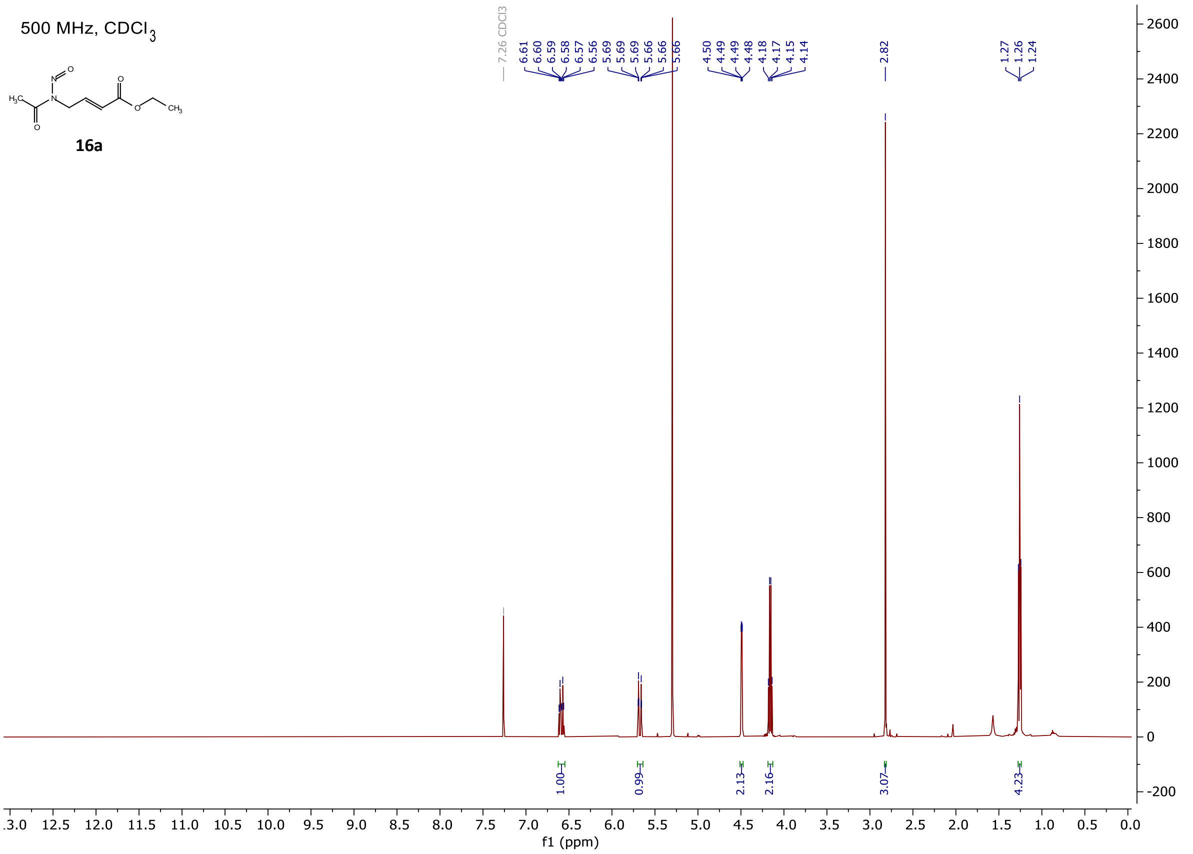
500 MHz, CDCl3
7.26 CDCl3
6.61
6.60
6.59
6.58
6.57
6.56
5.69
5.69
5.69
5.66
5.66
5.66
4.50
4.49
4.49
4.48
4.18
4.17
4.15
4.14
2.82
1.27
1.26
1.24
1.00
0.99
2.13
2.16
3.07
4.23
f1 (ppm)

16a

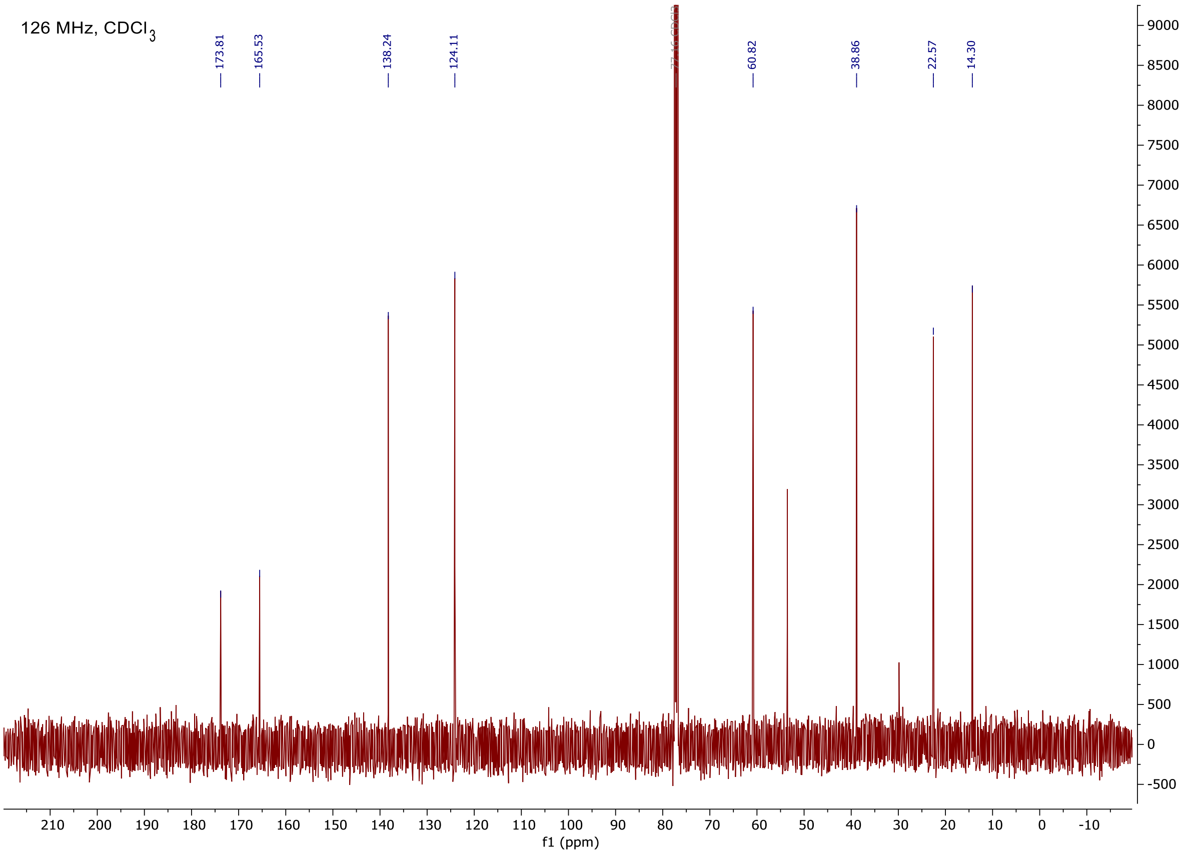

126 MHz, $CDCl_3$
173.81
165.53
138.24
124.11
60.82
38.86
22.57
14.30
f1 (ppm)

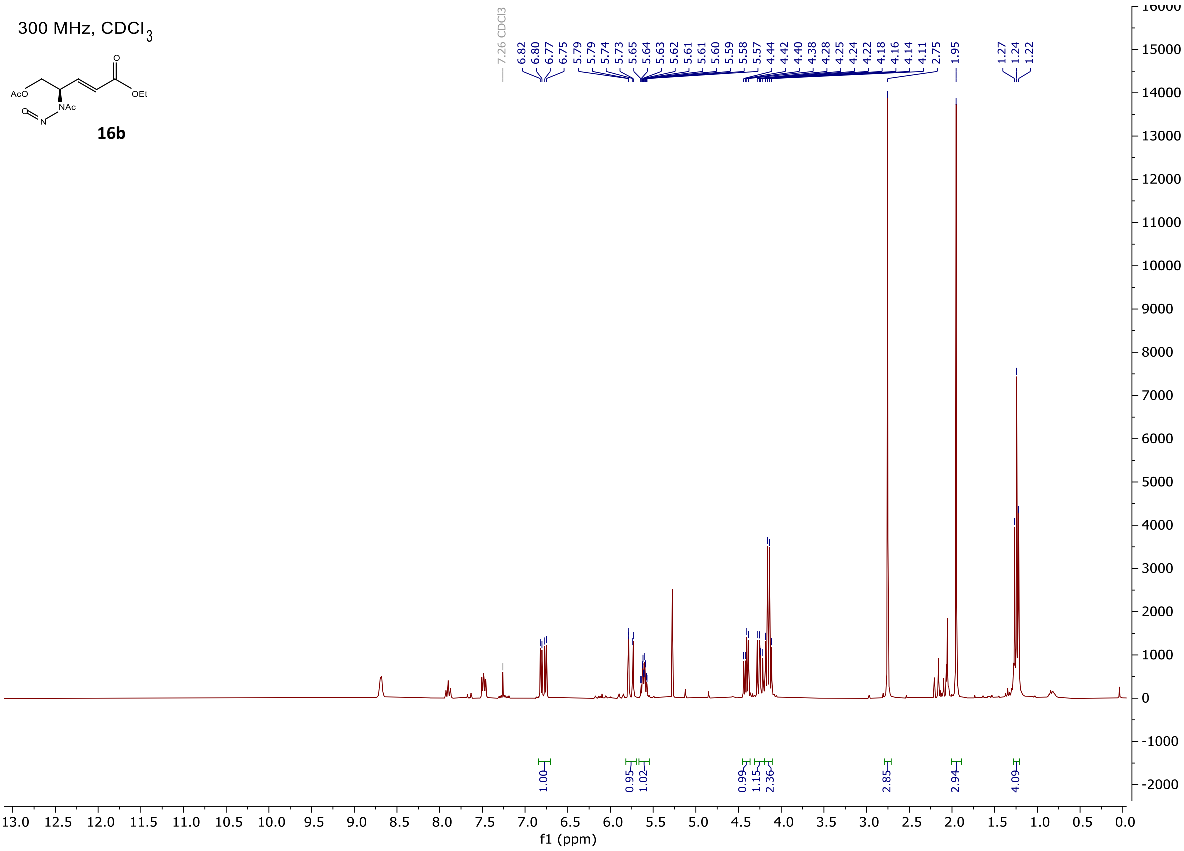

300 MHz, $CDCl_3$
AcO
NAc
OEt
O
N
16b
7.26 CDCl3
6.82
6.80
6.77
6.75
5.79
5.79
5.74
5.73
5.65
5.64
5.63
5.62
5.61
5.61
5.60
5.59
5.58
5.57
4.44
4.42
4.40
4.38
4.28
4.25
4.24
4.22
4.18
4.16
4.14
4.11
2.75
1.95
1.27
1.24
1.22
1.00
0.95
1.02
0.99
1.15
2.36
2.85
2.94
4.09
f1 (ppm)

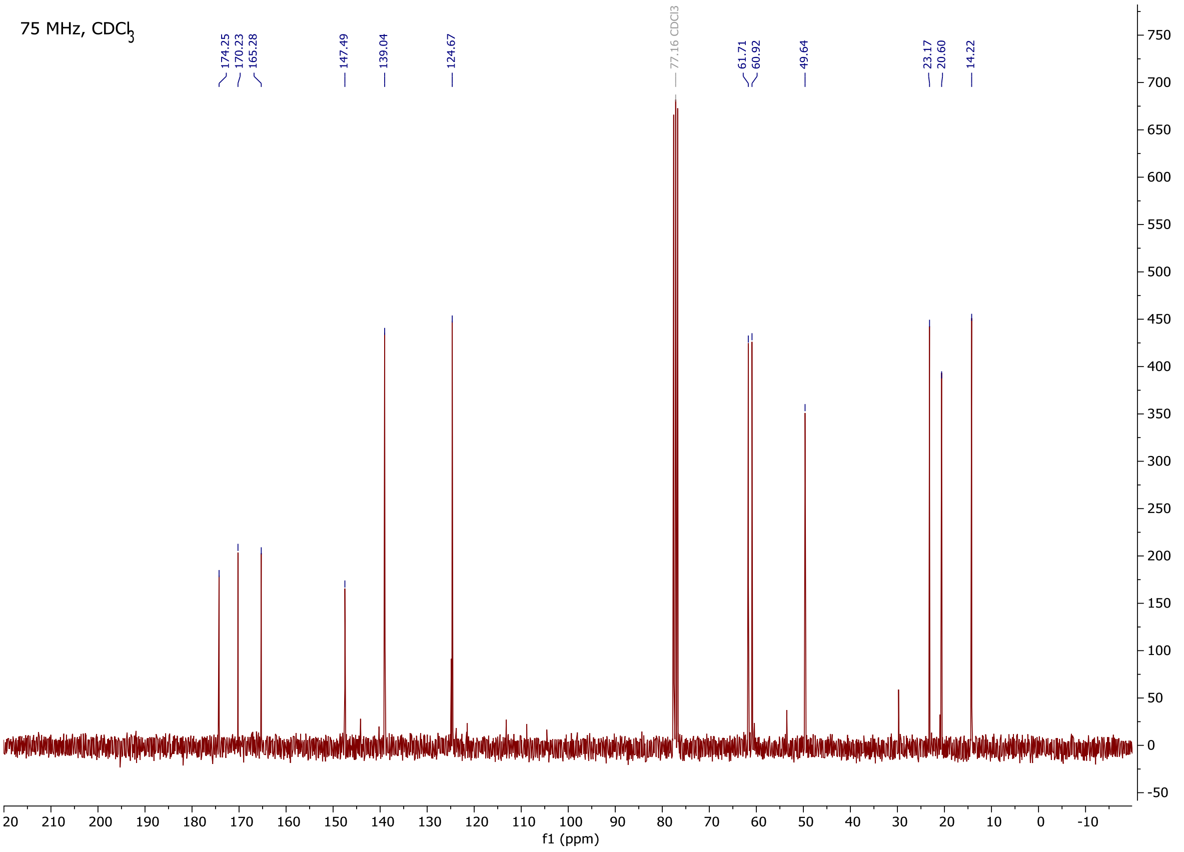
75 MHz, CDCl3
174.25
170.23
165.28
147.49
139.04
124.67
77.16 CDCl3
61.71
60.92
49.64
23.17
20.60
14.22
f1 (ppm)

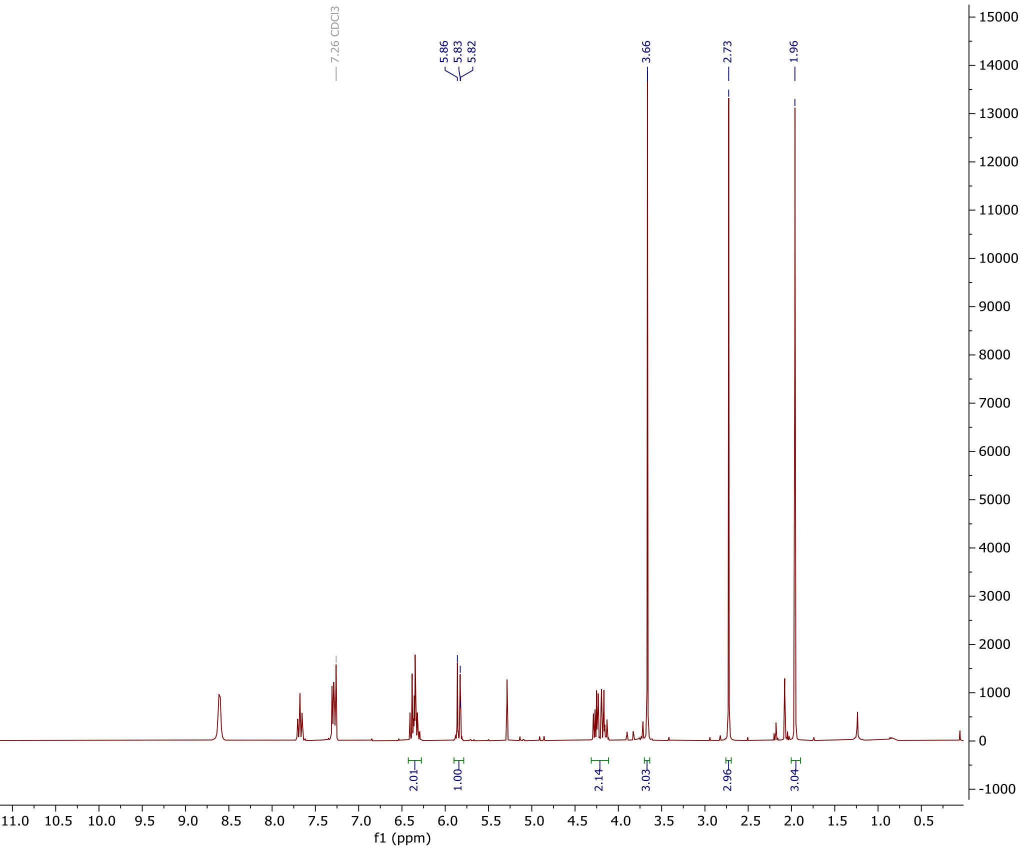
300 MHz, CDCl3
7.26 CDCl3
5.86
5.83
5.82
3.66
2.73
1.96
2.01
1.00
2.14
3.03
2.96
3.04
f1 (ppm)

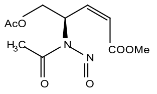
AcO
H3C
N
N
O
O
COOMe

16c

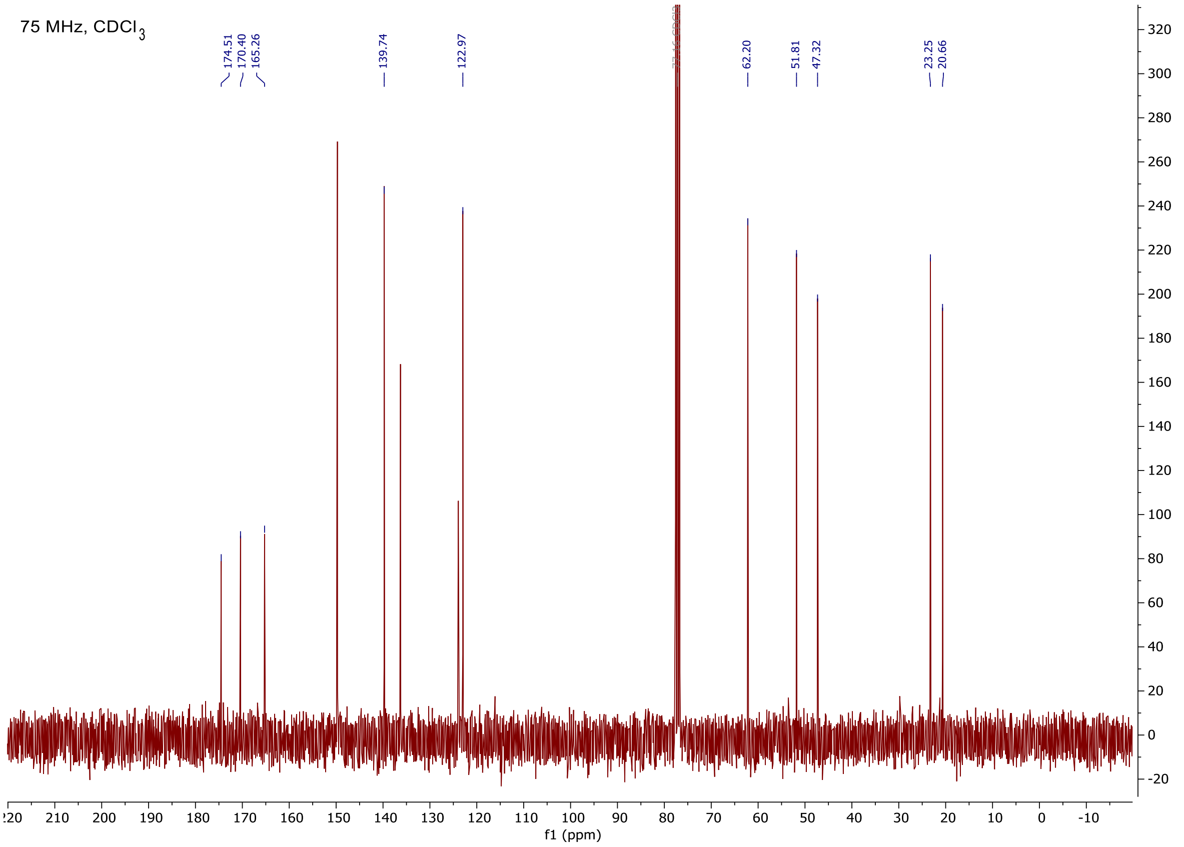
75 MHz, CDCl3
174.51
170.40
165.26
139.74
122.97
62.20
51.81
47.32
23.25
20.66
f1 (ppm)

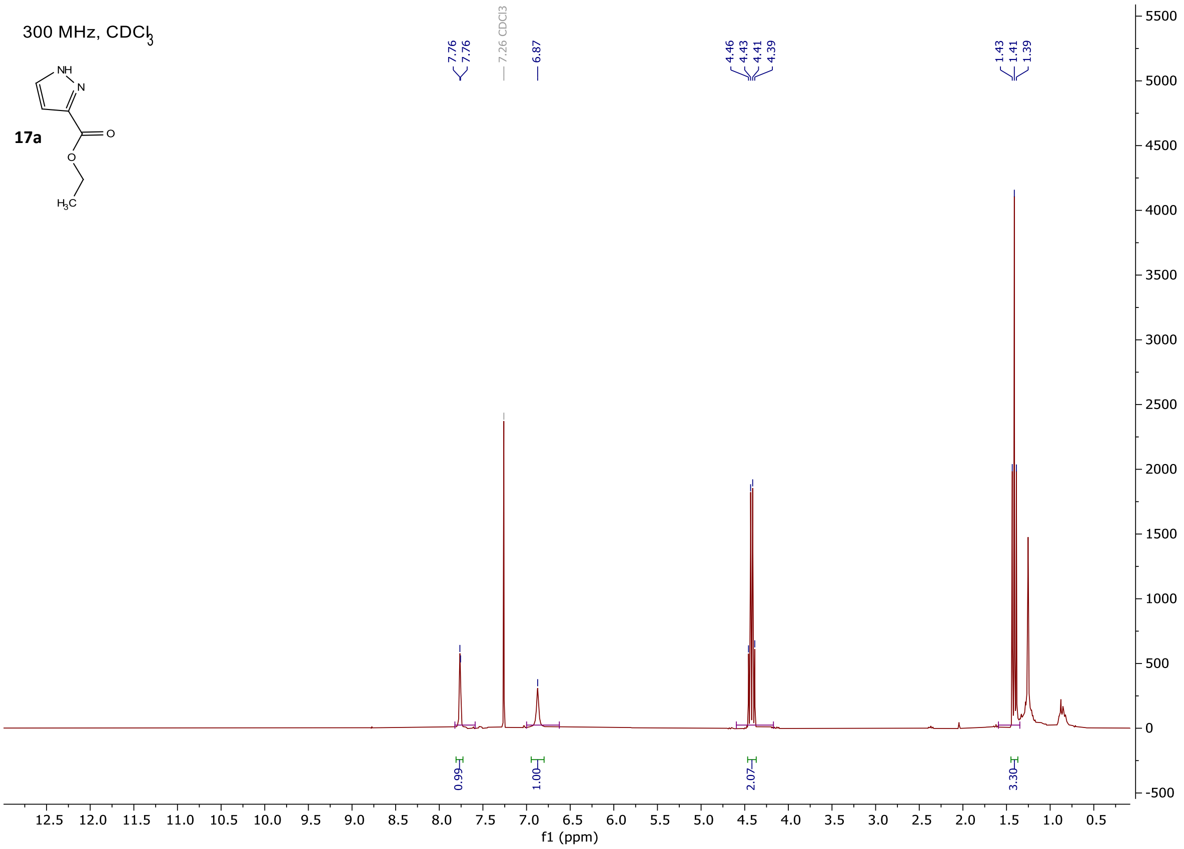
300 MHz, CDCl3
17a
7.76
7.76
7.26 CDCl3
6.87
4.46
4.43
4.41
4.39
1.43
1.41
1.39
0.99
1.00
2.07
3.30
f1 (ppm)

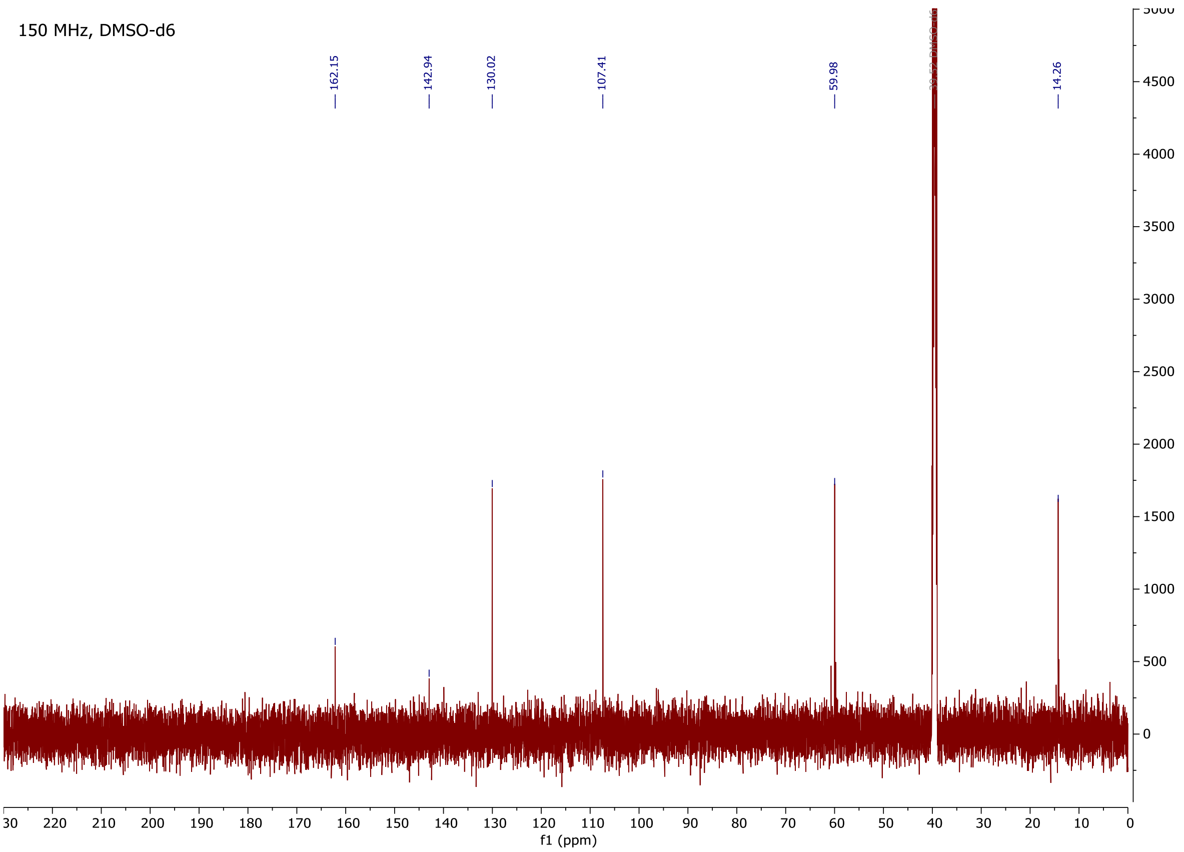
150 MHz, DMSO-d6
162.15
142.94
130.02
107.41
59.98
14.26
f1 (ppm)

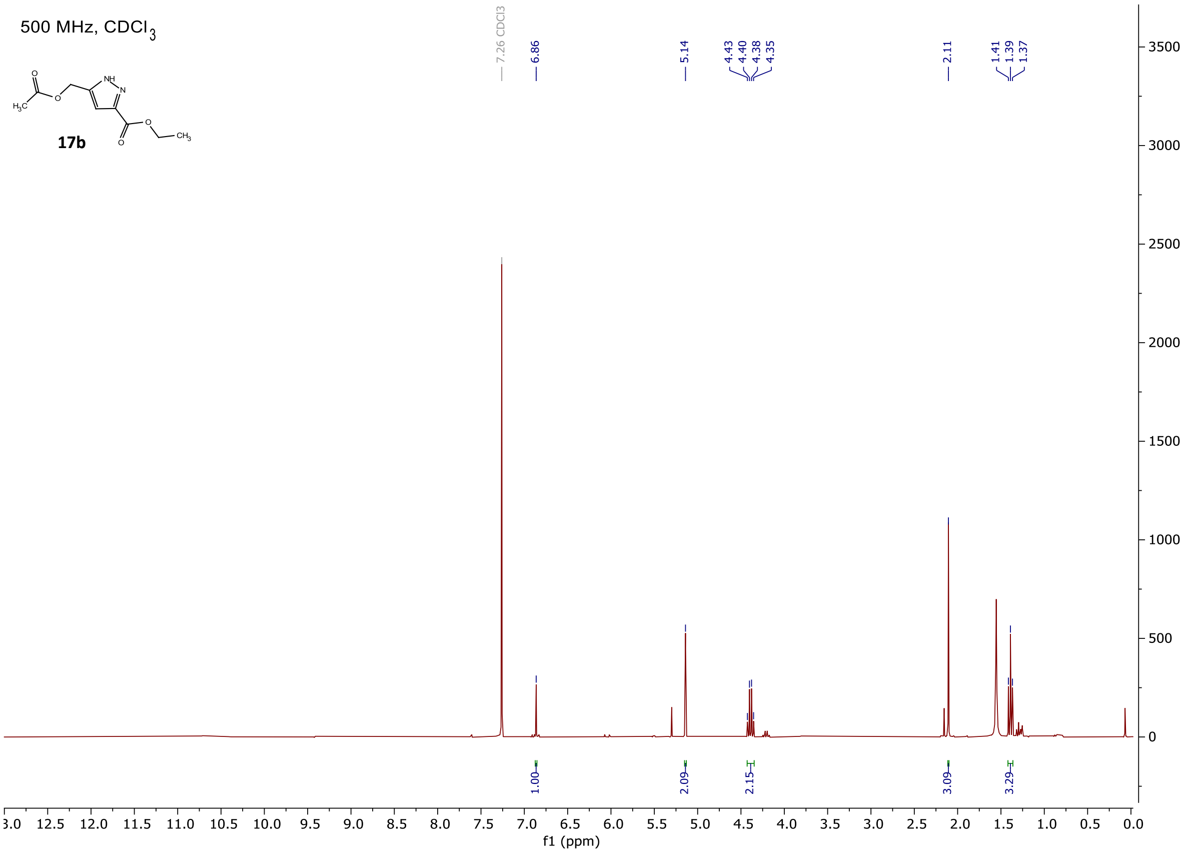
500 MHz, $CDCl_3$
17b
7.26 CDCl3
6.86
5.14
4.43
4.40
4.38
4.35
2.11
1.41
1.39
1.37
1.00
2.09
2.15
3.09
3.29
f1 (ppm)

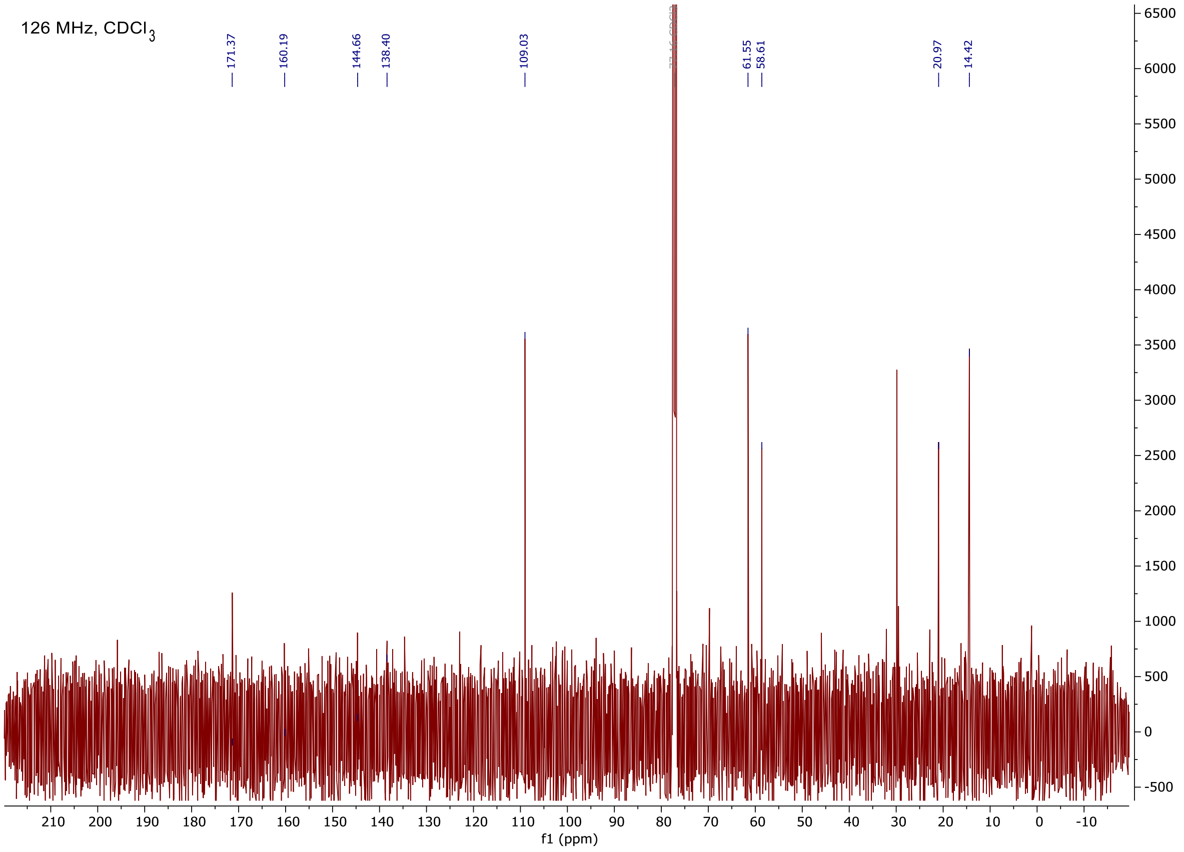
126 MHz, $CDCl_3$
171.37
160.19
144.66
138.40
109.03
61.55
58.61
20.97
14.42
f1 (ppm)

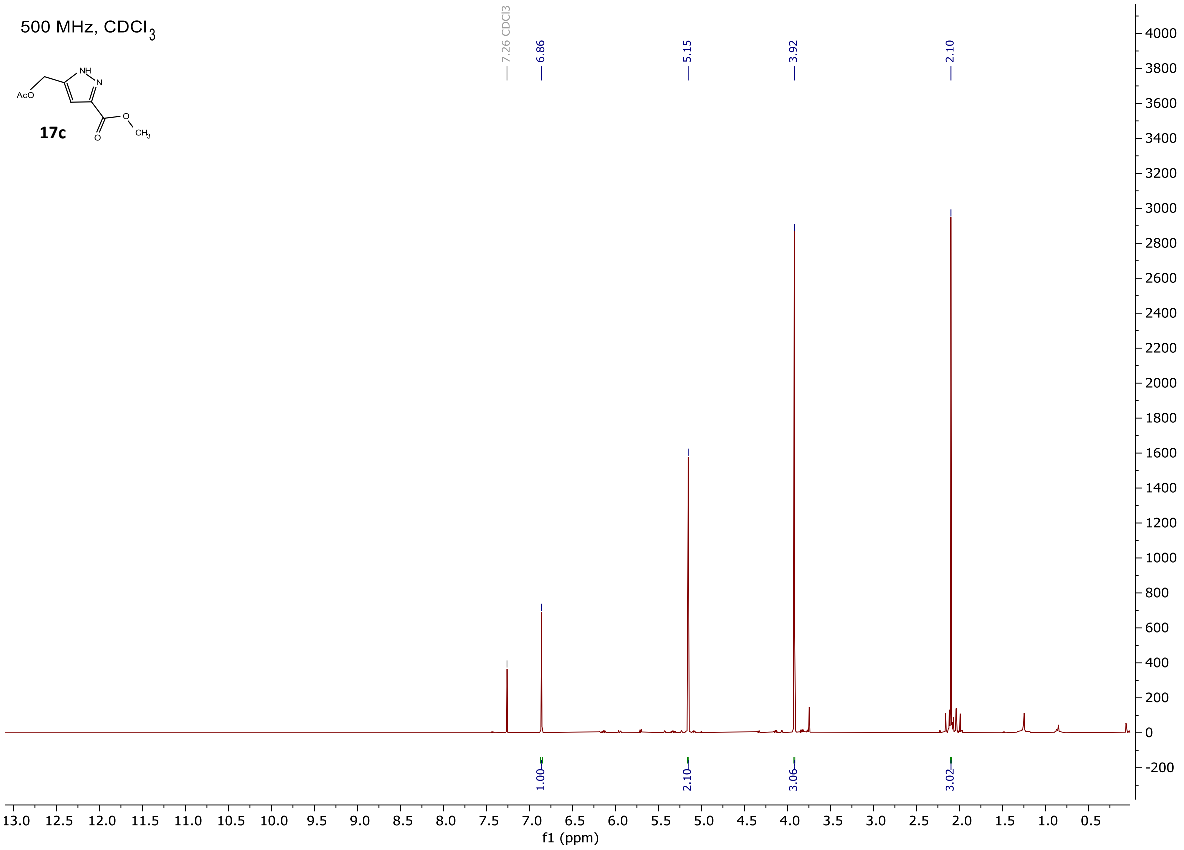
500 MHz, $CDCl_3$
AcO
NH
N
O
O
CH_3
17c
7.26 CDCl3
6.86
5.15
3.92
2.10
1.00
2.10
3.06
3.02
f1 (ppm)

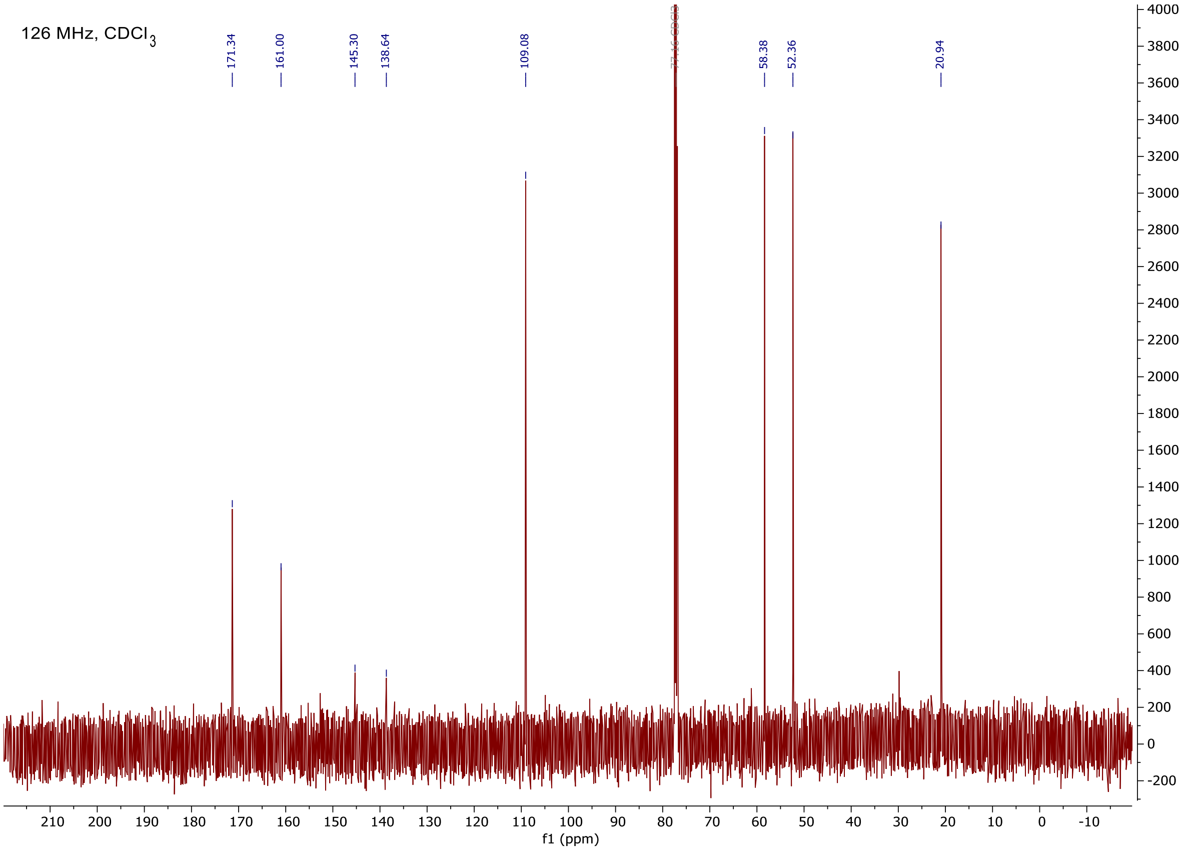
126 MHz, CDCl3
171.34
161.00
145.30
138.64
109.08
58.38
52.36
20.94
f1 (ppm)

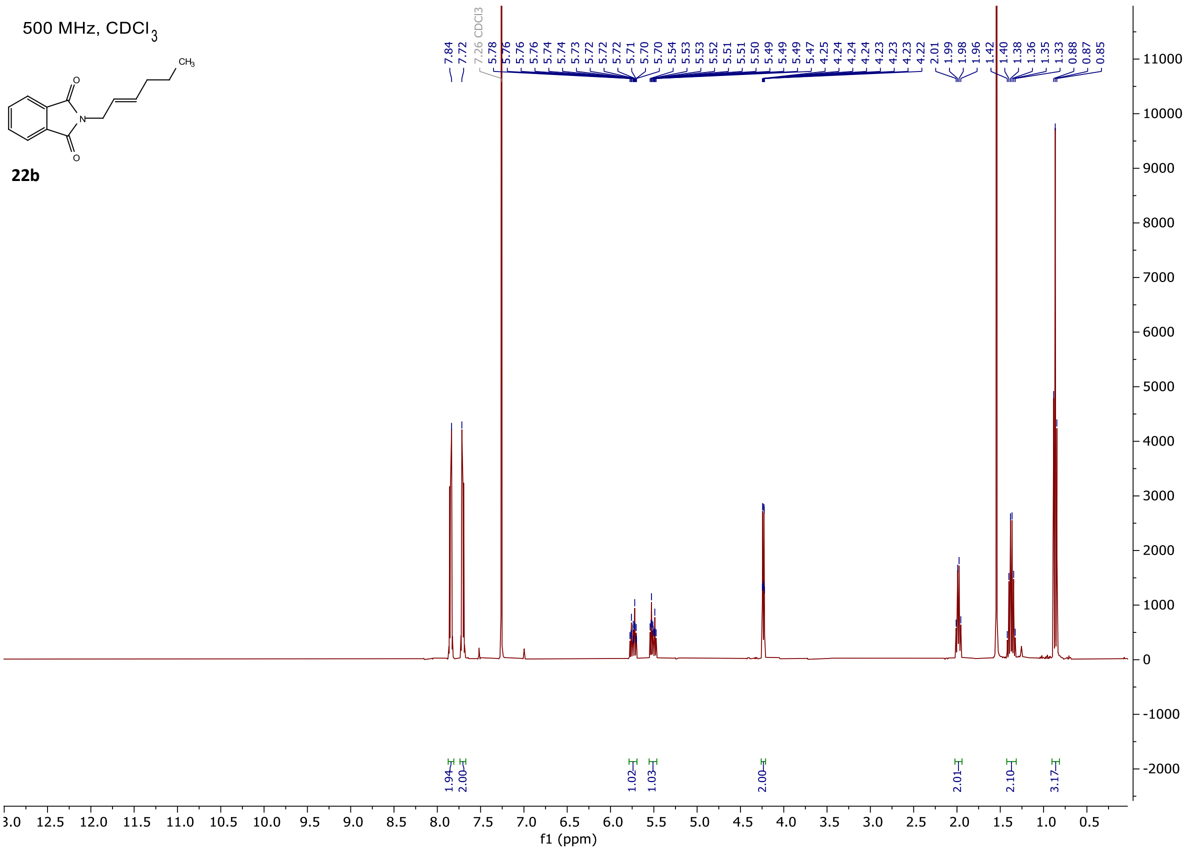
500 MHz, CDCl3
22b
7.26 CDCl3
f1 (ppm)
1.94
2.00
1.02
1.03
2.00
2.01
2.10
3.17

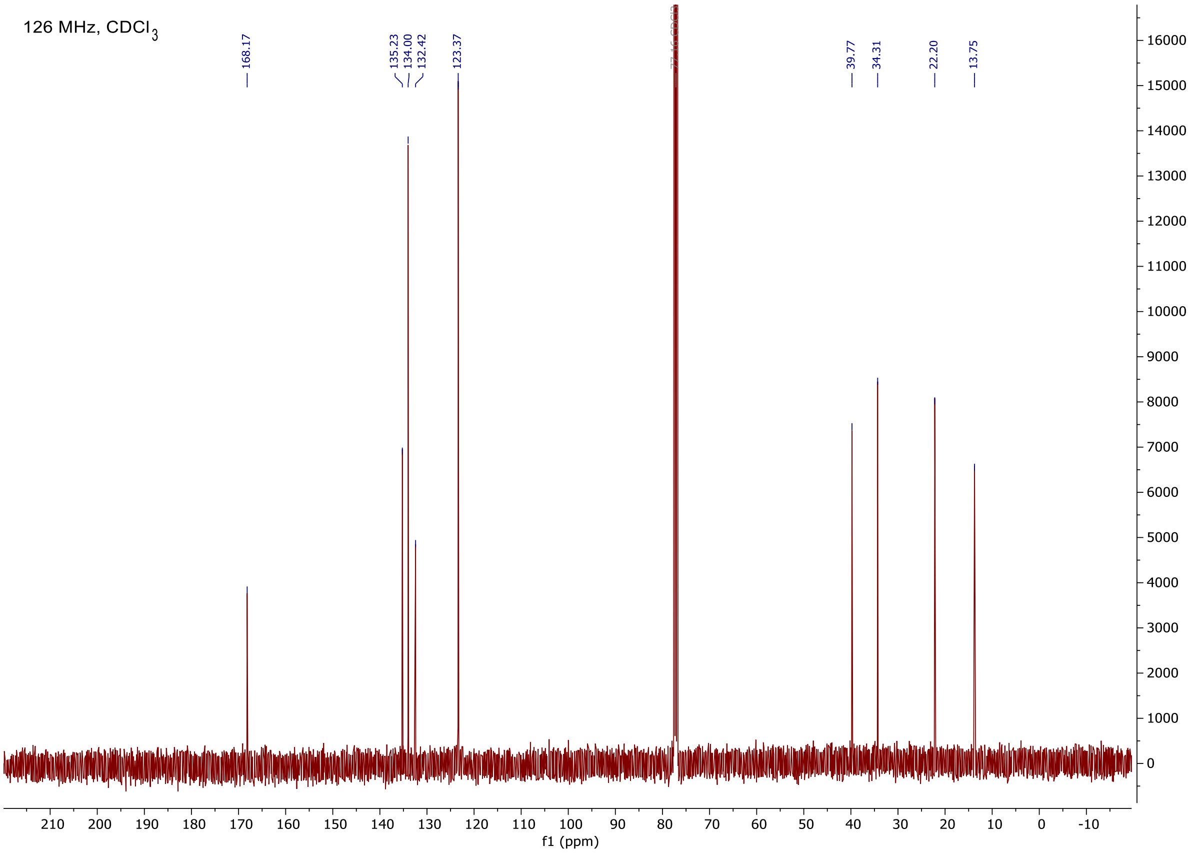
126 MHz, CDCl3
168.17
135.23
134.00
132.42
123.37
39.77
34.31
22.20
13.75
f1 (ppm)

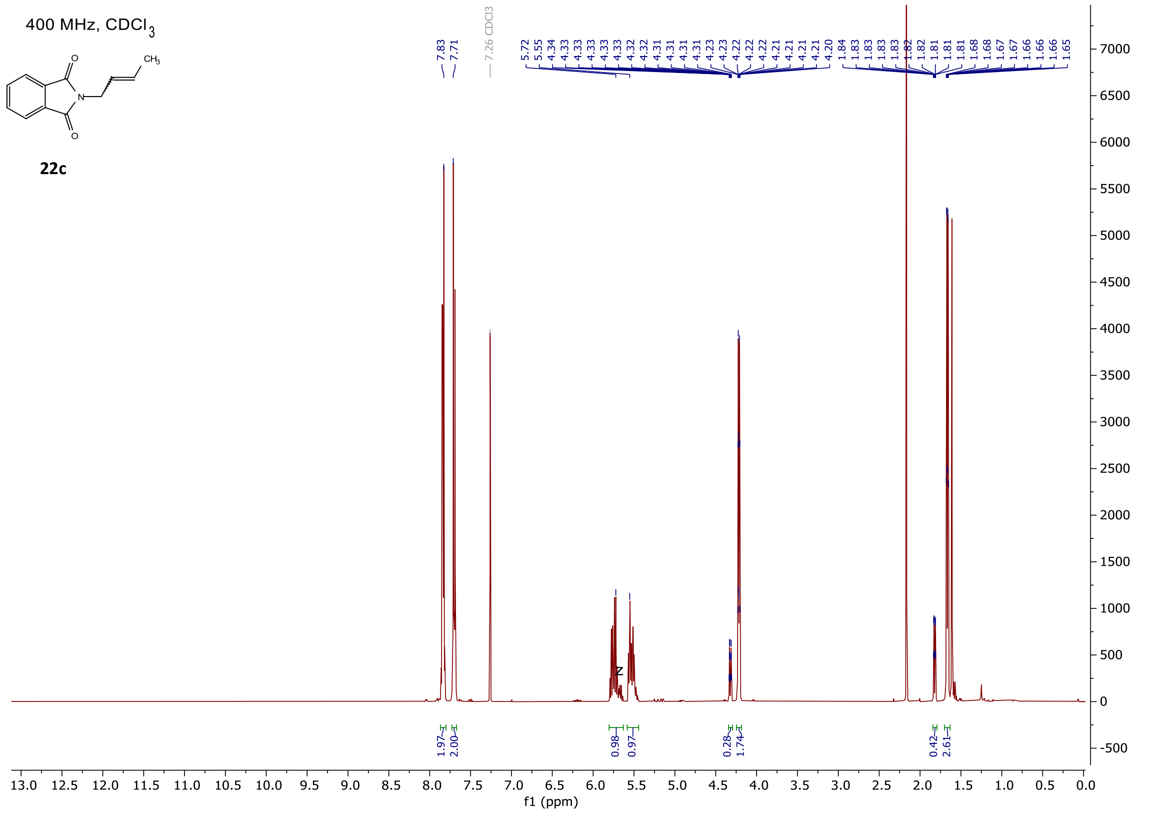
400 MHz, CDCl3
CH3
N
O
O
22c
7.83
7.71
7.26 CDCl3
5.72
5.55
4.34
4.33
4.33
4.33
4.33
4.33
4.32
4.32
4.31
4.31
4.31
4.31
4.23
4.22
4.22
4.22
4.21
4.21
4.21
4.21
4.20
1.84
1.83
1.83
1.83
1.82
1.82
1.81
1.81
1.68
1.68
1.67
1.66
1.66
1.66
1.65
Z
1.97
2.00
0.98
0.97
0.28
1.74
0.42
2.61
f1 (ppm)

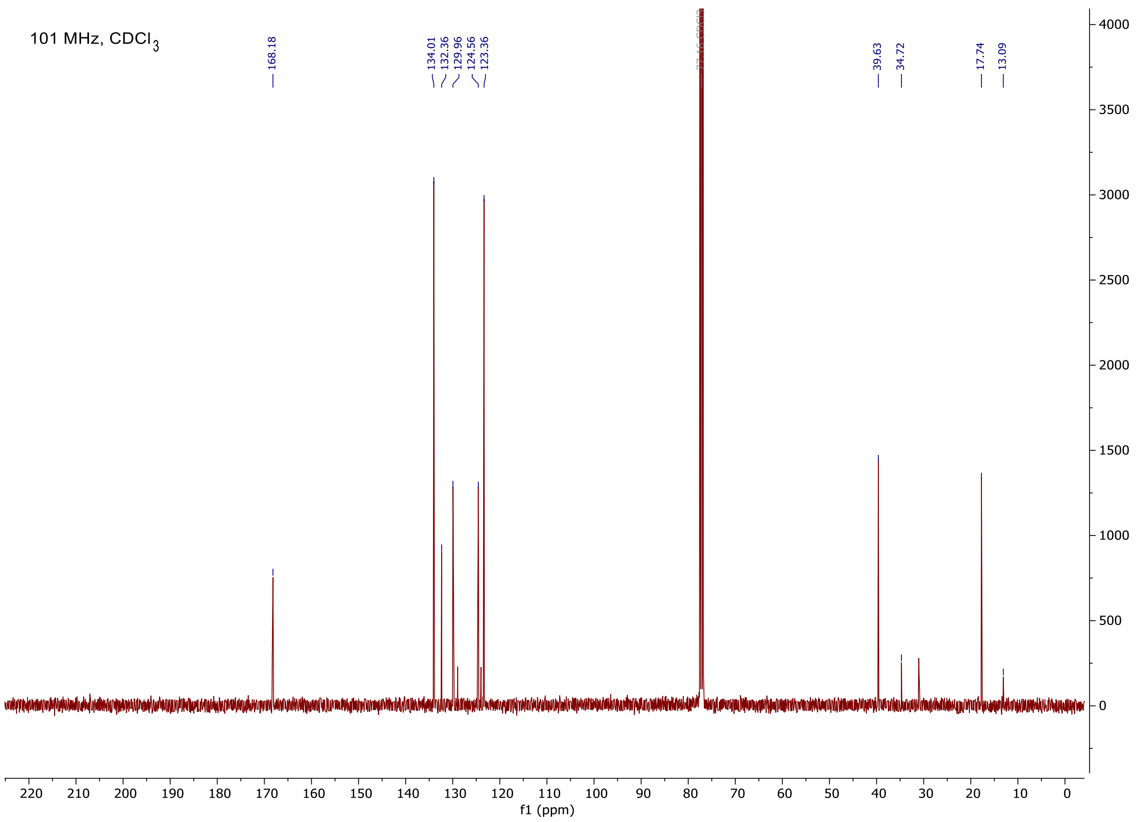
101 MHz, CDCl3
168.18
134.01
132.36
129.96
124.56
123.36
39.63
34.72
17.74
13.09
f1 (ppm)
